# Enhancing start-up and torque in Darrieus VAWTs through a novel plain gurney flap design

**DOI:** 10.1038/s41598-026-38485-9

**Published:** 2026-02-03

**Authors:** Wallaaldin Eltayeb, Jarupula Somlal, Mustafa SirElkhatim, G. Yedukondalu, A. Srinath

**Affiliations:** 1 Department of Electrical and Electronics Engineering, Koneru Lakshmaiah Education Foundation, Vaddeswaram, Guntur, Andhra Pradesh 522502 India; 2 Department of Chemical Engineering, Red Sea University, 3315 Port Sudan, Sudan; 3 Department of Mechanical Engineering, Koneru Lakshmaiah Education Foundation, Vaddeswaram, Guntur, Andhra Pradesh 522502 India

**Keywords:** Darrieus VAWT, Plain flap, Plain gurney flap, Torque enhancement, Self-starting wind turbine, Rural wind energy, Energy science and technology, Engineering

## Abstract

Enhancing aerodynamic efficiency and self-starting torque in Darrieus-type Vertical Axis Wind Turbines (VAWTs) is essential for power generation under low and intermittent wind conditions. This study presents a systematic evaluation of Plain Flap (PF), Gurney Flap (GF), and hybrid Plain Gurney Flap (PGF) modifications applied to the NACA 0015 aerofoil. Two-dimensional Unsteady Reynolds-Averaged Navier-Stokes (URANS) simulations using the SST k-ω model were conducted across a tip-speed-ratio (TSR) range of 0.2–4.5 and Reynolds numbers ((Re = 5.4 × 10^4^–2.7 × 10^5^), with results validated by prototype testing. The optimal 0.5c, 10° PF configuration demonstrated the most robust and stable performance across the full operational range. At low TSR (0.2–0.5), the PF significantly improved mean Cm by 30–40% and Cp by 40% by increasing effective camber. While the 0.015c PGF generated higher instantaneous torque, it incurred larger downwind losses. At moderate TSR (0.8–1.5), PF and PGF achieved similar high Cp gains (40–50%). Beyond TSR = 2.0, the PF maintained superior aerodynamic stability and consistent efficiency, whereas the PGF performance declined due to drag penalties. Prototype testing conclusively confirmed these numerical trends, with PF blades increasing shaft speed by up to 51% at 5.5 m/s compared to the baseline. Overall, PF and PGF configurations effectively mitigate weak self-starting and low efficiency, providing a validated passive-flap design pathway for efficient small-scale and hybrid solar-wind energy systems.

## Introduction

In 2024, the global wind industry is expected to continue its strong growth, following the record addition of 117 GW in 2023. Meeting the 1.5 °C climate target will require tripling annual installations to about 320 GW by 2030^1,2^. However, challenges such as energy storage remain, driving continued investment to improve the reliability and efficiency of wind power. This growth aligns with COP28 priorities and highlights the urgency of meeting global climate goals^2^. VAWTs are increasingly considered in cities and regions where wind availability is irregular, as they can harness flows from different directions^3–5^.

These turbines present a feasible solution for decentralised power in rural settings with limited wind resources. Although recent advances in aerodynamic design have improved their effectiveness, VAWTs still underperform compared with Horizontal Axis Wind Turbines (HAWTs) and often need external assistance for start-up^6–9^. Progress in computational modelling and aerodynamic optimisation is gradually closing this gap, enhancing their potential for both offshore deployment and rural electrification^10^.

With global energy demand rising and climate change pressures intensifying, VAWTs are emerging as a strong alternative in locations where HAWTs are less effective^11^. Their ability to capture wind from multiple directions makes them especially suitable for rural and variable-wind environments^6,12,13^.

The aerodynamic behaviour of VAWTs is strongly influenced by aerofoil geometry, particularly under low-Reynolds number (Re) and low-TSR conditions. Symmetric NACA profiles, such as 0015, 0018, and 0021, have been widely adopted in Darrieus turbines because they provide stable torque characteristics and maintain higher efficiency across a wide operating range^14^. In contrast, cambered profiles improve static torque but often suffer from reduced efficiency at higher TSRs, limiting their broader applicability^15^. Among symmetric profiles, the NACA 0015 has emerged as one of the most extensively studied aerofoils for VAWT applications. Its moderate thickness and proven aerodynamic stability under low- and medium-TSR operation have led to its frequent use in both Computational Fluid Dynamic (CFD) and experimental benchmarking^12,16,17^. Accordingly, the NACA 0015 is employed in this study as the reference profile for trailing-edge flap optimisation.

PFs are trailing-edge devices that effectively increase aerofoil camber, thereby enhancing lift and delaying flow separation at low TSRs. Numerous CFD and wind tunnel studies have shown that PFs can improve the self-starting capability of Darrieus turbines by increasing static torque and reducing negative torque dips. Singh^18^ demonstrated that a ± 15° deflection on a NACA 66 − 015 aerofoil improved lift and delayed stall by altering surface pressure distributions. More recently, Abed et al.^19^ confirmed that moderate deflections of 10° achieved the best balance between lift and efficiency, while larger angles introduced drag penalties.

Kaya^20^ tested hybrid PF-rib designs on a NACA 0018 aerofoil, raising maximum lift-to-drag ratio from 27.7 to 28.7. Gains were strongest at moderate to high Angle of attack, showing that morphing PF concepts can outperform conventional PFs. Parluhutan et al.^21^ also reported that while very high flap deflections (30°) maximised lift, they caused substantial drag increases, reducing the lift-to-drag ratio .

In the context of Darrieus turbines, PFs have been shown to improve self-starting performance and raise Cp at low TSRs, particularly when applied at mid-chord positions. However, the benefits diminish at higher TSRs, as flap-induced drag offsets lift gains. These findings highlight PFs as a simple and effective device for improving low-speed performance but also underline the importance of optimising flap position and deflection.

GFs are small, fixed tabs mounted at the trailing edge that enhance circulation and modify wake structure, thereby increasing lift and torque at low TSRs. Their benefits are most pronounced under low-Re conditions typical of small-scale turbines. Extensive research has been carried out on GFs to improve the aerodynamic efficiency of wind turbines^22^. Li et al.^23^ were the first to introduce the GF, showing that GFs with heights ranging from 1% to 5% of the chord length can significantly enhance aerofoil performance, particularly in VAWTs. Additionally, Xie et al.^24^ demonstrated that incorporating Improving the NACA 0012 aerofoil with a GF led to a 22% boost in energy capture and a 15% improvement in efficiency.

Additionally numerous research has investigated the impact of GFs on the capability of Darrieus VAWTs at low and moderate TSRs. Yan et al.^25^ observed that GFs enhance lift and reducing drag by producing trailing-edge vortices, leading to a substantial rise in Cp at reduced TSRs. Bianchini et al.^26^ reported up to 20% Cp improvements on a Darrieus turbine with NACA 0021 blades equipped with 2% chord-height GFs. Mousavi et al.^27^ similarly found that angled GF designs enhanced lift-to-drag ratio and reduced hinge moments, improving both aerodynamic and structural performance.

Deflection angle is also a critical parameter. Experimental studies by Pambudi et al.^28^ on outboard GFs confirmed that a 120° orientation outperformed the conventional 90°, raising Cp to 0.228 at TSR 1.166 and improving torque stability. In contrast, 90° GFs consistently increased drag and degraded torque stability, with reductions in average Cm of up to 36%. These results indicate that while GFs are structurally simple and effective at enhancing torque in weak winds, their geometry must be carefully tuned to avoid efficiency penalties.

The combination of PFs and GFs has recently been explored to exploit the complementary benefits of both devices. A PGF integrates a hinged PF with a small trailing-edge tab, thereby enhancing lift through camber modification while simultaneously improving circulation and delaying flow separation. Chen et al.^29^ reported that PGFs improved torque generation at low TSRs more effectively than either PFs or GFs alone, addressing one of the long-standing barriers of Darrieus turbines: poor self-starting capability.

The current literature presents a clear need for a focused, validated comparison of these passive flow control devices. While PFs, GFs, and PGFs have been studied individually, systematic comparison on the benchmark NACA 0015 aerofoil across multiple Reynolds number regimes, coupled with experimental validation, remains significantly limited. Most existing work relies solely on 2D CFD or steady-state analyses, often neglecting the dynamic stall and structural effects critical for real-world turbine operation.

This work directly addresses these gaps through a comprehensive evaluation of PF, GF, and hybrid PGF modifications applied to the NACA 0015 aerofoil. Our primary contributions are:

1. Systematic and comparative aerodynamic evaluation: We conducted comprehensive Unsteady RANS simulations using the SST K-$\:\omega\:$ model to systematically compare the effects of PF, GF, and hybrid PGF modifications on Cp, torque Cm, and start-up torque across a wide range of TSR (0.2–4.5) and Re (5.4 × 10^4^–2.7 × 10^5^).
2. Identification of optimal, robust configuration: We rigorously identified the 0.5, 10° PF as the optimal configuration, providing consistent aerodynamic gains across the entire low-to-medium TSR range and delivering the most balanced performance, while also maintaining simpler fabrication and implementation compared to PGF.
3. Experimental validation of aerodynamic trends: Numerical results were validated using prototype testing with a 1 m rotor, which verified the predicted aerodynamic trends and confirmed the practical applicability of the design for enhancing self-starting capability in low-wind, urban, and rural environments.

The results provide new aerodynamic insight into how passive trailing-edge modifications enhance the efficiency and self-starting capability of Darrieus VAWTs, offering critical design guidance for small-scale turbines.

## Computational framework

### Domain setup and boundary parameters

**Fig. 1:**
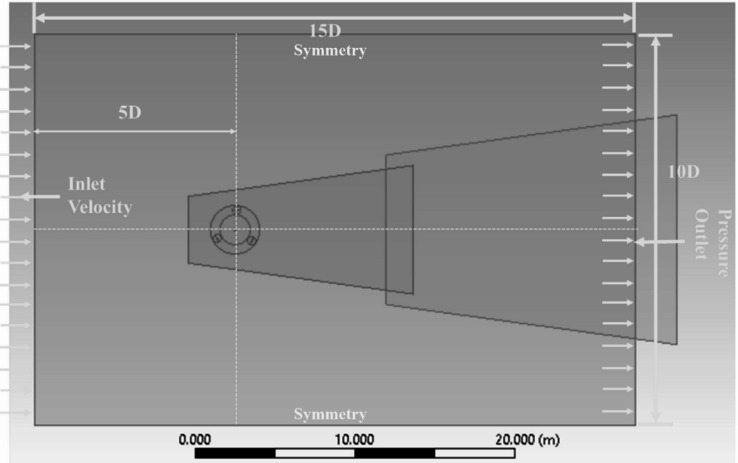
Simulation domain.

**Fig. 2:**
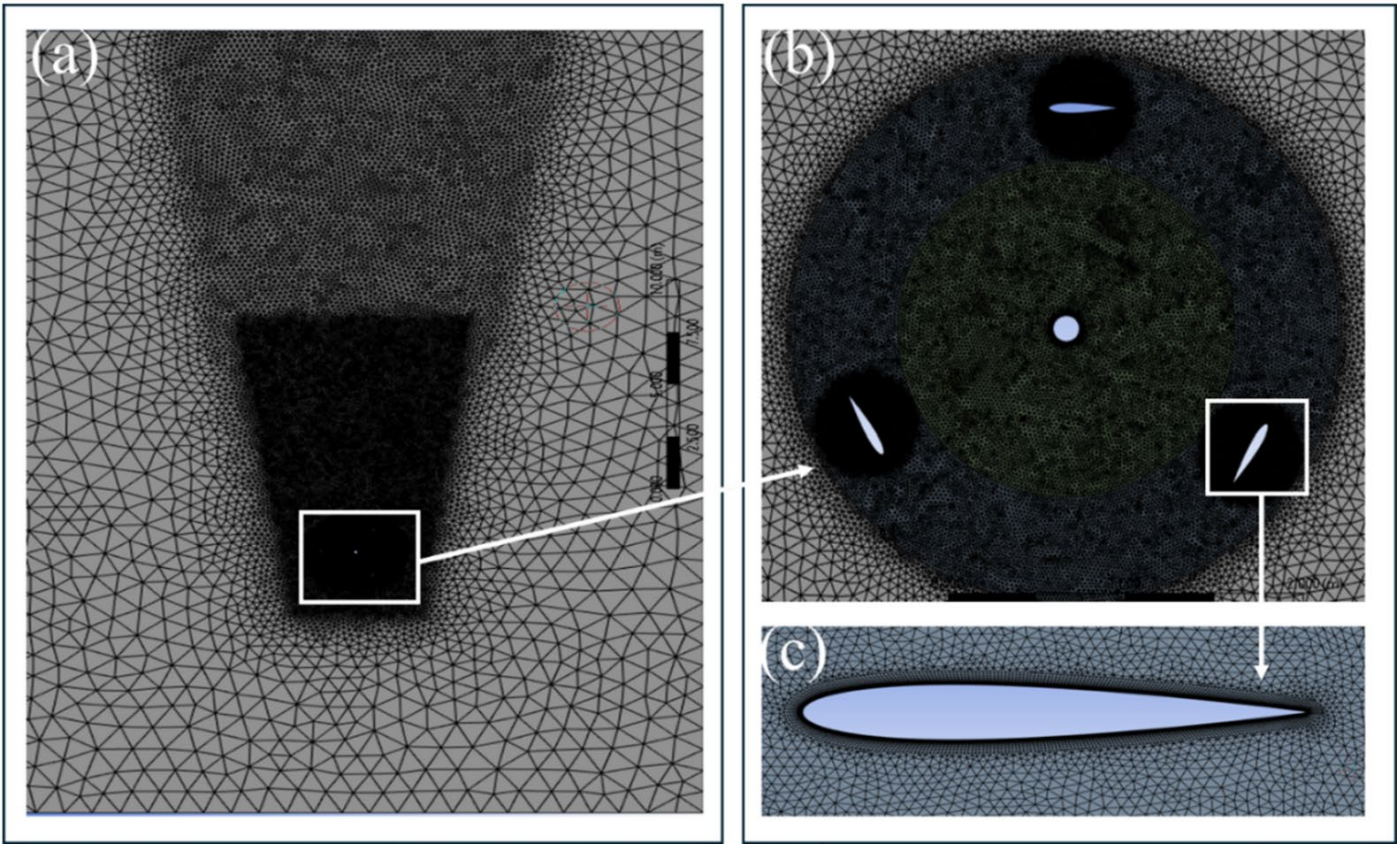
Numerical grid: (**a**) Stationary domain mesh, (**b**) Mesh surrounding the rotating, inner, and shaft, (**c**) Mesh surrounding aerofoil.

The study employs a carefully optimized computational domain to precisely replicate the intricate flow dynamics of Darrieus VAWT^17^. As depicted in Fig. 1, The domain has a shaft (0.04D), fixed zone (15D), rotating region (1.5D), and inner zone (0.75D). The top and lower boundaries are placed 10D distant to ensure adequate wake formation while eliminating boundary effects. To ensure stable inflow, the upstream boundary is positioned 5D away, while the downstream boundary is placed 10D further, acting as a pressure outlet to facilitate effective wake flow dispersion and minimize domain effects. Figure 2 illustrates the computational mesh around the domain and the aerofoil. Figure 2a highlights the domain mesh, Fig. 2b focuses on the mesh within the rotating domain, and Fig. 2c zooms in on the mesh around the aerofoil, emphasizing boundary layer refinement.

A sliding mesh interface facilitated interaction between the moving and fixed domains, ensuring precise flow exchange for analysing turbine rotation. The boundary conditions included velocity inlets, slip boundaries to simulate open-air flow, an outlet for pressure release, and no-slip boundaries on the blades and shaft to mimic airflow behaviour. This study simulates small-scale wind turbine operation under air density of 1.225 kg/m^3^ and viscosity of 1.7894 × 10^− 5^ kg/m s, with inlet velocities of 5 m/s and 10 m/s. The setup considers Re from 5.4 × 10^4^ to 2 × 10^6^ and TSRs between 0.5 and 4.5 to replicate real conditions.

Table 1 demonstrates the essential variables utilised in the study, elucidating the Darrieus VAWT’s structural attributes and the CFD model. It outlines key aspects including baseline aerofoil, length of the chord, diameter of the rotor, number of blades, solver type, model of turbulence, among other critical details. These specifications ensure reliable simulations and accurate results, providing an extensive description of the analysis and design of turbines.

**Table 1:** Specifications of the turbine and parameters used in CFD simulations.

Specifications of the turbine	Details
Blade type	NACA 0015
Length of the chord (C)	0.16 m and 0.4 m
The diameter of the rotor (D)	1.0 m and 2.5 m
Power rating	1.4 kilowatts and 3.5 kilowatts
Blade count	Three blades
Entry velocity (V)	Flow velocities of 5 m/s and 10 m/s.
Re	5.4 × 10^4^ and 2.71 × 10^5^
TSR	Varies between 0.5 to 4.5

### Selection of geometric parameters

The Darrieus wind turbine was optimized by extensive CFD simulations. These simulations concentrated on key geometric parameters, especially the position of the PF along the blade, spanning from 10% to 90% of the Length of the Chord. The PF deflection angle was fixed at 10° based on previous numerical optimisation results reported by^22^. In that study, flap deflections of 10° and 20° were compared for NACA 2412 and NACA 0015 airfoils, and the 10° configuration yielded superior aerodynamic performance, producing higher Cm and Cp with reduced drag. The 20° case led to premature separation and lower efficiency. Therefore, a 10° deflection was adopted in the present work as the most effective and validated configuration for improving turbine performance under low-Re conditions. In addition, GF heights varying from 0.015c to 0.025c were evaluated with different degrees of deflection of 90° and 120°. The combination of PF and GF, as shown in Table 2, includes the configuration of 0.5c at 10° paired with 0.025c at 120°. Research conducted by Chakroun and Bangga^30^ revealed that a GF positioned at 2% of the chord length with a 120° deflection angle significantly increased Cp. Table 2 provides a detailed breakdown of the aerofoil designs, specifying the PF and GF locations. Additionally, the PGF configuration, which integrates a 0.5c, 10° PF with a 0.015c, 120° GF and a 0.025c, 120° GF, is included to assess its combined aerodynamic effects.

**Table 2:**
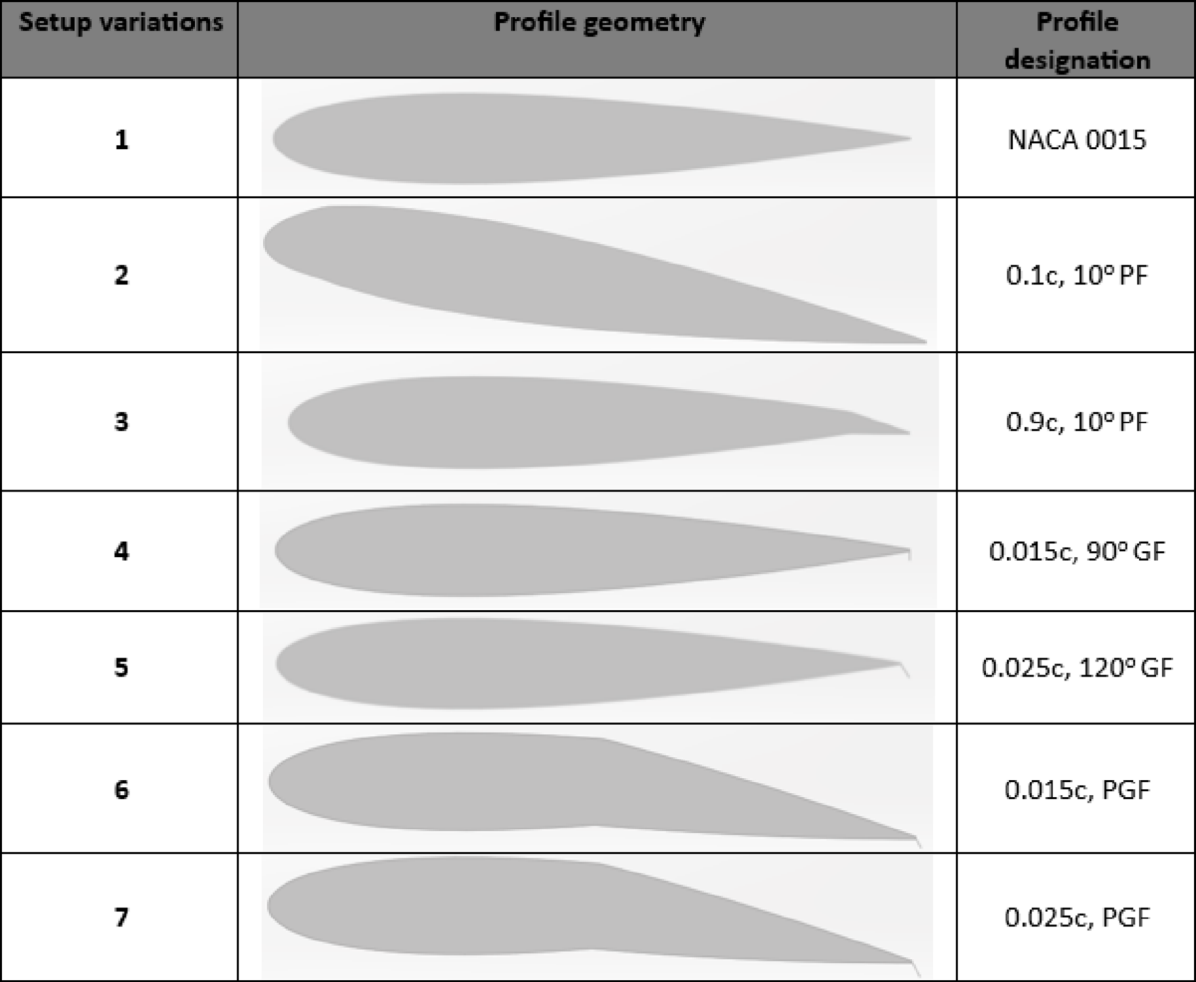
Configurations of plain, gurney, and hybrid flaps on the NACA 0015 profile.

### Mesh independence analysis

**Fig. 3:**
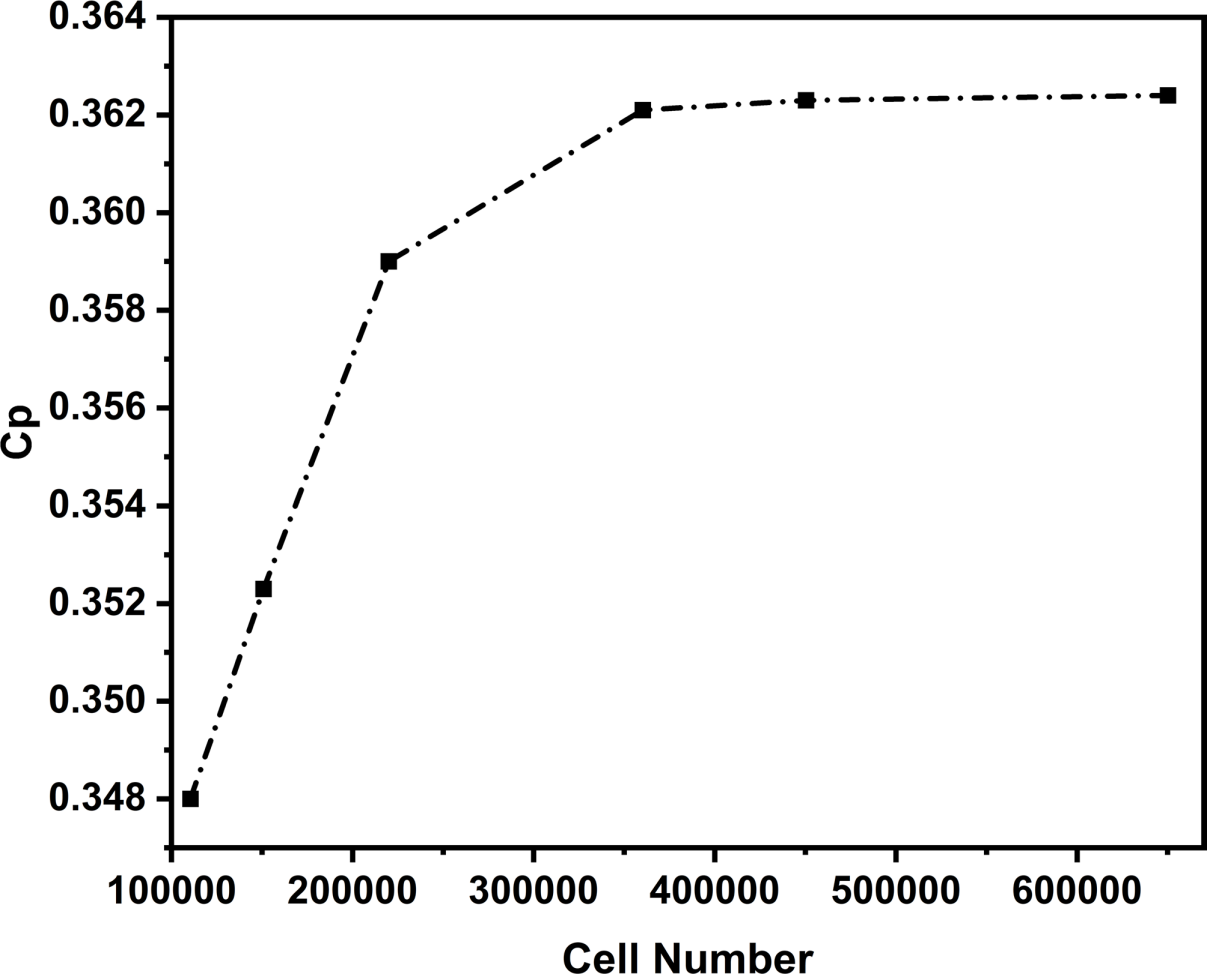
Evaluation of grid independence.

The domain was discretised with a structured inflation mesh to resolve the boundary layer. Five grids, 110,520–650,210 cells, were evaluated at TSR = 2.5 to balance accuracy and cost. Results are shown in Fig. 3 and summarised in Table 3. The coarsest grid (110,520 cells) deviated by 3.9% in Cp from the finest mesh (650,210 cells). The medium grid (360,220 cells) achieved Cp = 0.3621 with 0.09% deviation, so we selected this configuration (Grid 4, ≈ 360,220 cells) for all simulations.

Near-wall resolution was checked with a y⁺ sweep (1.5–6). Grid 4 used 15–20 inflation layers with a 1.2–1.25 growth rate and a first-layer height of 1.0 × 10^− 4^ m, giving y⁺ ≈ 3.1 at U = 10 m/s (Re ≈ 2.7 × 10^5^). Mesh quality met accepted criteria: average orthogonal quality 0.96, maximum skewness 0.79, maximum aspect ratio 97.4. These settings ensured stable convergence and accurate SST k–ω wall treatment.

**Table 3:** NACA 0015 aerofoil mesh independence analysis.

Grid size	Element count	Average y+	Cp
Grid 1	110,520	6	0.348
Grid 2	150,810	5.6	0.3523
Grid 3	220,000	4.7	0.359
Grid 4	360,200	3.1	0.3621
Grid 5	450,312	2.4	0.3623
Grid 6	650,210	1.5	0.3624

## Numerical techniques

### Performance parameters

Wind energy utilization efficiency is affected by the TSR-dependent energy deficit^31^. TSR is calculated as the ratio of the speed at the blade’s tip to the undisturbed wind speed, where R is the radius of the rotor, ω is the turbine’s angular velocity, and V_in_ ​ is the inlet velocity.

1 $$\:TSR\:\left(\lambda\:\right)=\frac{R\times\:\omega\:}{{V}_{in}}$$

The turbine’s energy extraction efficiency is evaluated using the Power Coefficient (Cp), defined as the ratio of the mechanical power output (P) to the available wind power passing through the rotor swept area (A)^32,33^:

2 $$\:{C}_{P}=\frac{P}{{P}_{wind}}=\frac{{T}_{\omega\:}}{\frac{1}{2}\rho\:A{V}_{\infty\:}^{3}}$$

where $\:\rho\:$ is the air density, $\:{V}_{\infty\:}$ is the free-stream wind velocity, T is the rotor torque, and $\:\omega\:$ is the angular velocity^34^.

The Cm​ equation for a wind turbine is generally expressed as^35^:

3 $$\:{C}_{m}=\frac{T}{\frac{1}{2}\rho\:AR{V}_{\infty\:}^{2}}$$

Here, T represents the torque generated by the turbine.

### The turbulence model and governing equation

This section outlines mathematical formulations and frameworks applied in the simulation, combined for clarity and consistency. Below is the continuity equation applicable to compressible and unstable flow conditions.

4 $$\:\frac{d\left(\rho\:{v}_{y}\right)}{d\mathrm{x}}+\frac{d\left(\rho\:{v}_{y}\right)}{dy}+\frac{d\left(\rho\:{v}_{v}\right)}{d\mathrm{z}}+\frac{d\rho\:}{d\mathrm{t}}=0$$

The fluid velocities in the directions along the x, y, and z axes are denoted as, $\:{v}_{x}$, $\:{v}_{y}$, and $\:{v}_{z}$​, correspondingly, with $\:\rho\:$ representing fluid density.

Momentum equation is:

5 $$\:\frac{d{(U}_{i)}}{dt}+{U}_{j}\frac{d{U}_{i}}{d{x}_{j}}=\frac{1}{\rho\:}\frac{dp}{d{x}_{i}}+\left[v\left(\frac{d{U}_{i}}{d{x}_{j}}+\frac{d\left({U}_{j}\right)}{d{x}_{i}}\right)-\stackrel{-}{{u}_{i}{u}_{j}}\right]$$

In this context, U_i_ and U_j_ represent time-averaged flow velocities, and $\:v$ indicates kinematic viscosity, balancing inertial, pressure, and viscous forces.

The kinetic energy conservation equation can be expressed in the form of:

6 $$\:\frac{d}{dt}\left(\rho\:k\right)+\frac{d}{d{x}_{j}}\left[\rho\:{u}_{j}k-\left(\mu\:+{\sigma\:}_{k}{\mu\:}_{t}\right)\frac{dk}{d{x}_{j}}\right]={\tau\:}_{tij}{S}_{ij}-\beta\:\mathrm{*}\rho\:\omega\:k$$

The rate of Omega’s specific consumption is represented by the following equation:

7 $$\:\frac{d}{dt}\left(\rho\:\omega\:\right)+\frac{d}{d{x}_{j}}\left[\rho\:{u}_{j}k-\left(\mu\:+{\sigma\:}_{\omega\:}{\mu\:}_{t}\right)\frac{d\omega\:}{d{x}_{j}}\right]={P}_{\omega\:}-\beta\:\rho\:{\omega\:}^{2}+2\left(1-{F}_{1}\right)\frac{\rho\:\sigma\:{\omega\:}_{2}}{\omega\:}\frac{dkdw}{d{x}_{i}d{x}_{i}}$$

Representing the eddy viscosity as µ_t_​, these formulas represent the behaviour of the energy of turbulence (k) and its corresponding rate of dissipation (ω).

### Solver setup

URANS simulations were performed using the SST k-ω turbulence model to evaluate the aerodynamic performance of the Darrieus VAWT. The SST k-ω model was selected for its numerical stability and proven accuracy across the Re considered in this study. Two operating regimes were analysed: a low-Re case (Re ≈ 5 × 10^4^) for the 1 m rotor at 5 m/s and a higher-Re case (Re ≈ 2.7 × 10^5^) for the 2.5 m rotor operating at 5–10 m/s. This model has been validated in previous VAWT studies^17,36,37^ for its ability to capture flow separation, dynamic stall, and wake-blade interactions. Although the Transition SST model can better capture laminar-turbulent transition, its high sensitivity to near-wall meshing and greater computational cost make it less practical for unsteady simulations. Pressure-velocity coupling was achieved using the SIMPLE (Semi-Implicit Method for Pressure-Linked Equations) algorithm, and all simulations were carried out in ANSYS Fluent 18.2 to ensure numerical stability.

Convective terms were discretised using a second-order upwind scheme to enhance spatial accuracy^37,38^. The computational domain was divided into rotating and stationary regions, with the blades positioned in the rotating zone to reproduce the local flow field around the rotor.

Transient simulations were advanced using a fixed angular increment defined by $\:\varDelta\:t\:=\frac{\varDelta\:\theta\:}{\omega\:}$. For the 1 m rotor operating at 5 m/s and TSR = 1.5, an increment of 0.2° per step resulted in Δt = 2.31 × 10^− 4^ s. For the 2.5 m rotor at 10 m/s, a 1° increment produced Δt = 1.45 × 10^− 3^ s at TSR = 1.5 and Δt = 8.72 × 10^− 4^ s at TSR = 2.5. The complete set of angular velocities, rotational speeds, and corresponding time-steps for all TSR cases is presented in Table 4. These values align closely with those adopted in earlier studies^17,36,39^, confirming the temporal resolution’s suitability for capturing unsteady aerodynamic effects in low-TSR Darrieus simulations.

Temporal convergence was verified following the guidelines reported by Rezaeiha, Montazeri and Blocken and Zhang et al.^17,40^. Angular increments of 2°, 1°, and 0.5° were tested at TSR = 1.5, and the results confirmed that the 1° increment provided sufficient temporal resolution and numerical stability for all subsequent simulations. This approach is consistent with validated time-step recommendations for unsteady Darrieus VAWT analyses in the literature.

The Courant–Friedrichs–Lewy (CFL) number was maintained within 0.4–0.9 across the domain, governed by local cell sizes of 0.02–0.05 m in the wake and outer flow regions. The near-wall first-layer height (1.0 × 10^− 4^ m) satisfied the y⁺ requirement and did not influence the maximum CFL value. Each time step required 20–25 inner iterations until all residuals dropped below 10^− 6^, confirming temporal convergence in line with (Rezaeiha et al., 2019).

**Fig. 4:**
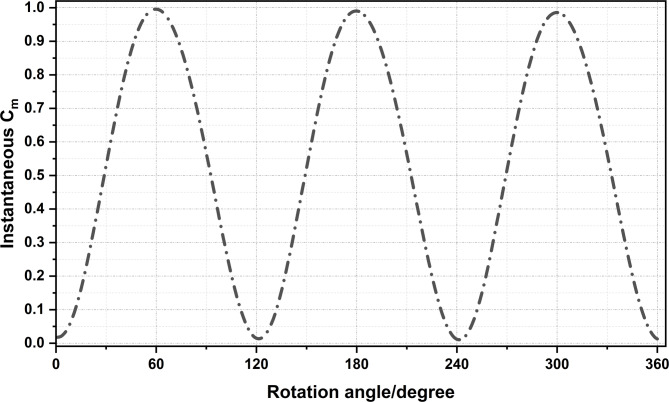
Variation of instantaneous Cm with blade angle for the NACA 0015 aerofoil at TSR = 2.5 (post-convergence).

The simulations were continued until periodic stability was achieved, typically after nine full revolutions, and performance data were extracted from the final cycle (Fig. 4).

The present model was solved in two dimensions, representing the mid-span section of the rotor. While 3D effects such as tip losses and root vortices were not explicitly modelled, their influence was minimised by extending the domain and applying the SST k–ω model, which reliably predicts separation and reattachment behaviour in low-Re VAWTs. This 2D approximation, commonly used in aerodynamic studies at small scale, effectively captures the dominant unsteady mechanisms governing torque and power generation. Experimental validation confirmed that the predicted performance trends matched observed behaviour, supporting the adequacy of the 2D approach for this analysis.

**Table 4:** Simulation parameters for different TSR at 10 m/s Inlet velocity for a 2.5 m diameter Darrieus wind turbine (1° time-step per iteration).

TSR	$\:{\omega\:}$ (rad/s)	Δt (1°) (s)
0.2	1.6	0.0109
0.5	4	0.00436
0.8	6.4	0.00273
1	8	0.00218
1.5	12	0.00145
2	16	0.00109
2.5	20	0.00087
3.5	28	0.00062
4.5	36	0.00048

## Validation analysis

### Mesh convergence verification

The results of the grid independence study, which are detailed in “Mesh independence analysis”, show that Grid 4, comprising 360,210 cells, as the most suitable option, balancing precision and computational cost. This configuration was utilized in the simulations to validate the CFD model and ensure an accurate depiction of flow behaviour around the turbine. The validation results confirm that this mesh configuration effectively supports the model’s capability to predict aerodynamic performance with high reliability.

### Verification

To assess the accuracy of the CFD simulations, results were compared with experimental data from Bravo et al.^41^. The study involved a 2D VAWT model operating with the inflow speed set to 10 m/s, giving a Re of 2 × 10^6^, consistent with previous research^17,42–45^.

**Fig. 5:**
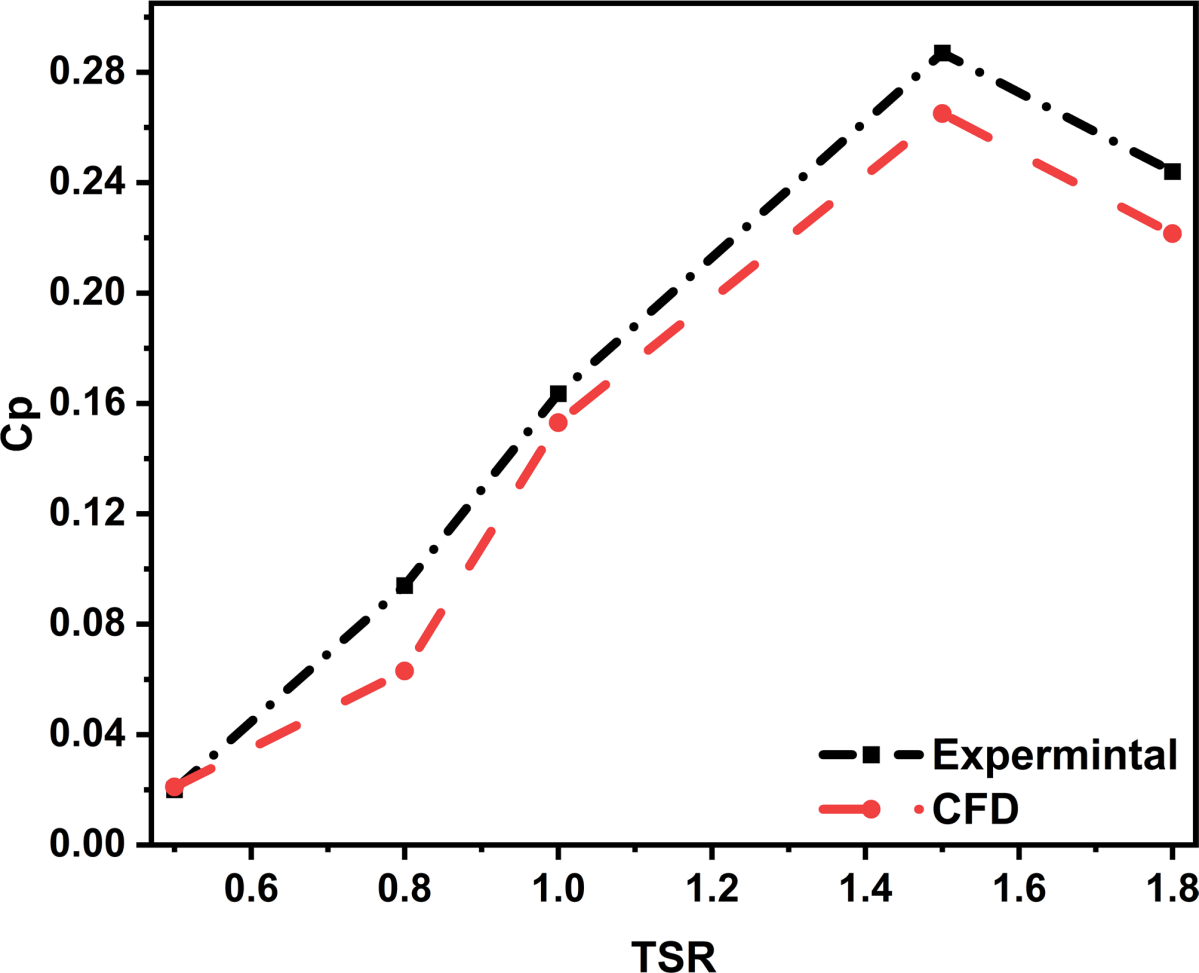
Comparison of present simulation outcomes against experimental data reported by Bravo et al.^41^.

Figure 5 illustrates the comparison of Cp values obtained from CFD simulations and experimental measurements across various TSRs. Figure 5 provides the comparison, highlighting that CFD simulations tend to overpredict Cp​ at lower TSRs, with the maximum deviation observed at TSR = 0.8, resulting in a percentage error of approximately 33%. This overprediction is attributed to the limitations of 2D modelling, which cannot capture three-dimensional phenomena such as upstream shear, tip vortex formation, and blade-root interactions.

However, the agreement between CFD and experimental data improves significantly at moderate to high TSRs (1.0–1.8), with percentage errors ranging from 6% to 9%. This indicates that the CFD model more accurately captures the turbine’s aerodynamic behaviour under stable flow conditions.

Similar trends have been reported in previous validated studies, where 2D URANS simulations yielded slightly lower Cp values than experiments due to the absence of full 3D flow features^9,46,47^. These consistent findings confirm that the present CFD framework provides physically realistic predictions within the expected range of accuracy for 2D aerodynamic analyses of Darrieus VAWTs.

To enhance the accuracy of future studies, it is recommended to implement 3D simulations and adaptive mesh refinement techniques to resolve complex flow structures more effectively, particularly at lower TSRs.

## Result and discussion

This section presents the aerodynamic performance of Darrieus VAWTs with passive TEF configurations, aimed at mitigating low self-start capability, torque ripple, and reduced Cp at low TSRs. Conventional designs include the PF, modelled in nine configurations with chordwise positions ranging from 10% to 90% and a fixed 10° deflection, and the GF, assessed in six variants with heights of 0.015c–0.025c at 90° and 120°. The advanced PGF combines the advantages of to boost lift while limiting drag penalties.

The NACA 0015 aerofoil is evaluated using Cp, Cm, torque ripple, and static starting torque, capturing both steady-state and unsteady performance. Table 2 summarises the geometric configurations of PF, GF, and PGF applied to the NACA 0015 aerofoil.

### Performance of plain flaps

**Fig. 6:**
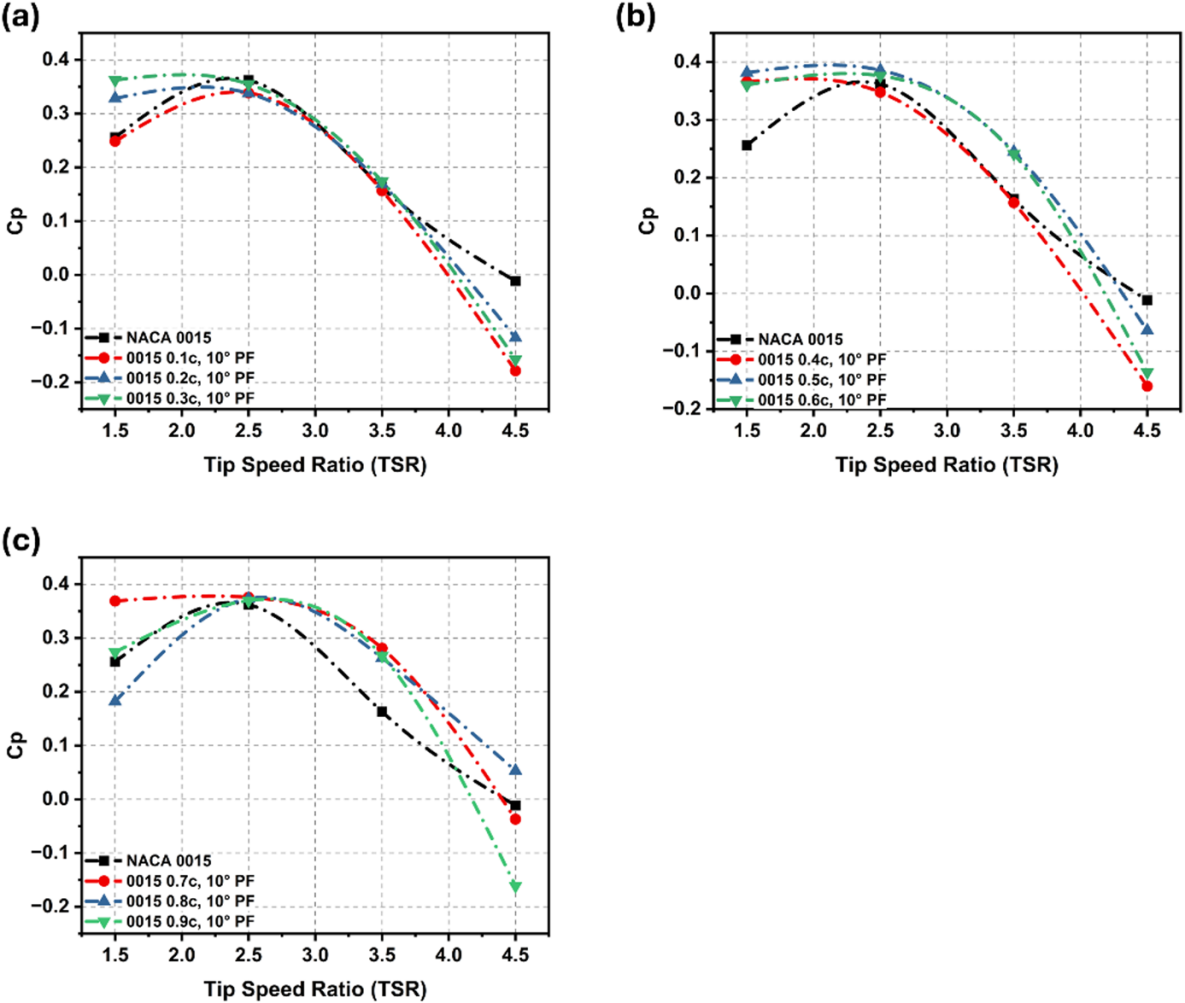
Cp comparison for flapless NACA 0015 aerofoil with PF configurations at 10° across various TSRs.

The aerodynamic performance of the PF configurations was evaluated across varying chordwise positions (0.1c–0.9c), as illustrated in Fig. 6a–c. Mid-chord configurations (0.4c–0.6c) demonstrated superior performance at low-to-moderate rotational speeds (TSR 1.5–2.5). Specifically, the 0.5c PF consistently achieved the highest efficiency, recording a Cp of 0.382 at TSR 1.5, an improvement of nearly 49.2% over the baseline (0.256). At TSR 2.5, this configuration maintained a robust lead with a 6.5% increase in Cp. In contrast, leading-edge (0.1c–0.2c) and near-trailing-edge (0.8c–0.9c) placements proved less effective in this regime, yielding negligible gains due to limited influence on flow reattachment^3^.

As the turbine transitioned to higher rotational speeds (TSR $\:\ge\:$ 3.5), the optimal flap position shifted aft. The 0.7c PF emerged as the most efficient design at TSR 3.5, producing a Cp of 0.281, which represents a 72.4% increase over the flapless rotor (0.163). This substantial gain is attributed to enhanced lift generation and delayed separation in the aft region of the blade. At the extreme condition of TSR 4.5, where most configurations suffered significant efficiency losses, only the near-trailing-edge flaps (0.8c-0.9c) sustained positive power coefficients, demonstrating superior aerodynamic stability under high-speed conditions. Overall, the 0.5c position offers the best balance for general operation, excelling in the critical low-to-medium TSR range, while the 0.7c position provides specific advantages for high-speed stability.

**Fig. 7:**
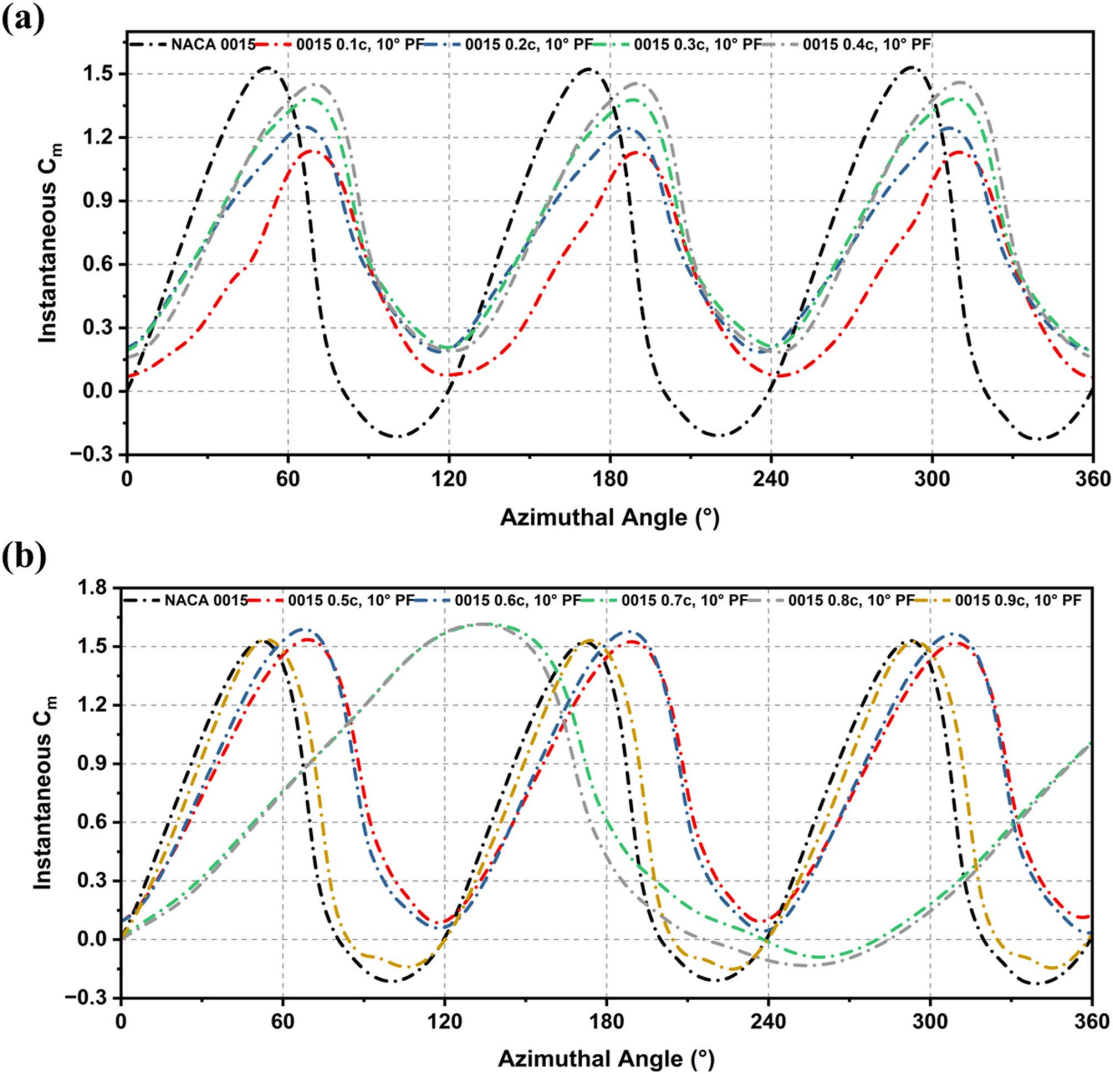
Instantaneous Cm over a complete rotation (0–360°) for NACA 0015 and flap-modified variants: (**a**) 0.1c to 0.4c, 10° deflection, (**b**) 0.5c to 0.9c, 10° deflection, under TSR = 1.5.

The influence of PF modifications on the instantaneous and average driving moment (Cm), a direct indicator of self-starting capability was analysed at Re = 2 × 10^6^ (2.5 m diameter, 10 m/s velocity). As illustrated in Fig. 7, all PF variations outperformed the baseline NACA 0015. Specifically, at TSR = 1.5, the 0.5c PF produced the most stable torque profile by enhancing positive peaks while simultaneously suppressing adverse negative dips. While trailing-edge placements (0.6c–0.7c) generated higher peak values, they induced deeper negative excursions, indicating a trade-off between torque amplification and stall sensitivity. Leading-edge flaps (0.1c–0.2c) provided only negligible gains.

**Fig. 8:**
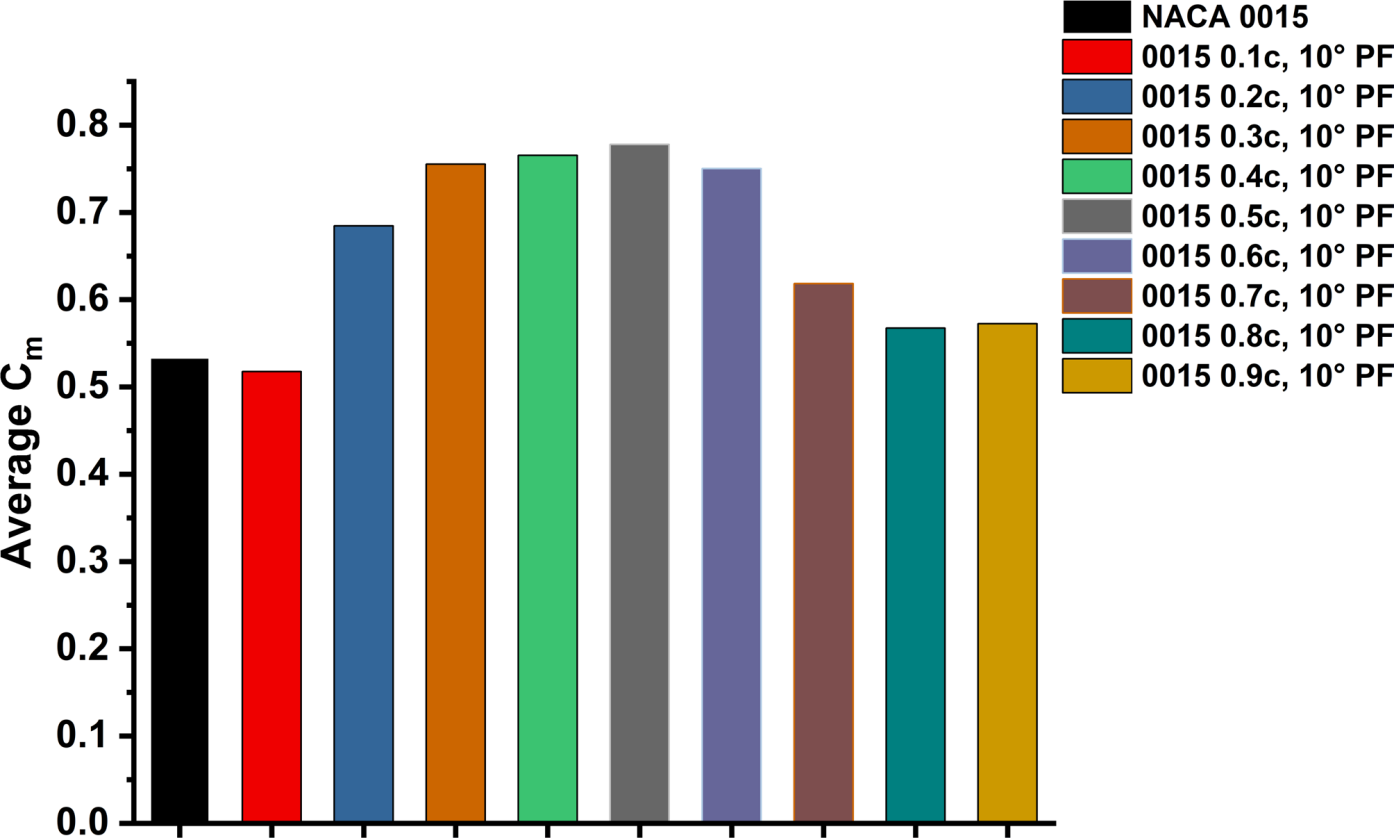
Average Cm for NACA 0015 and PF configurations (0.1c to 0.9c) at 10° deflection, under TSR = 1.5.

Average Cm trends (Fig. 8) confirm the superiority of mid-chord placements. The 0.5c configuration achieved an average Cm of 0.778, a 45.9% increase over the baseline (0.533). The 0.3c and 0.4c configurations also demonstrated strong performance, with improvements of 41% and 43%, respectively. These results identify the 0.5c PF as the most robust configuration for enhancing both start-up torque and steady-state operation under high-Re conditions.

**Fig. 9:**
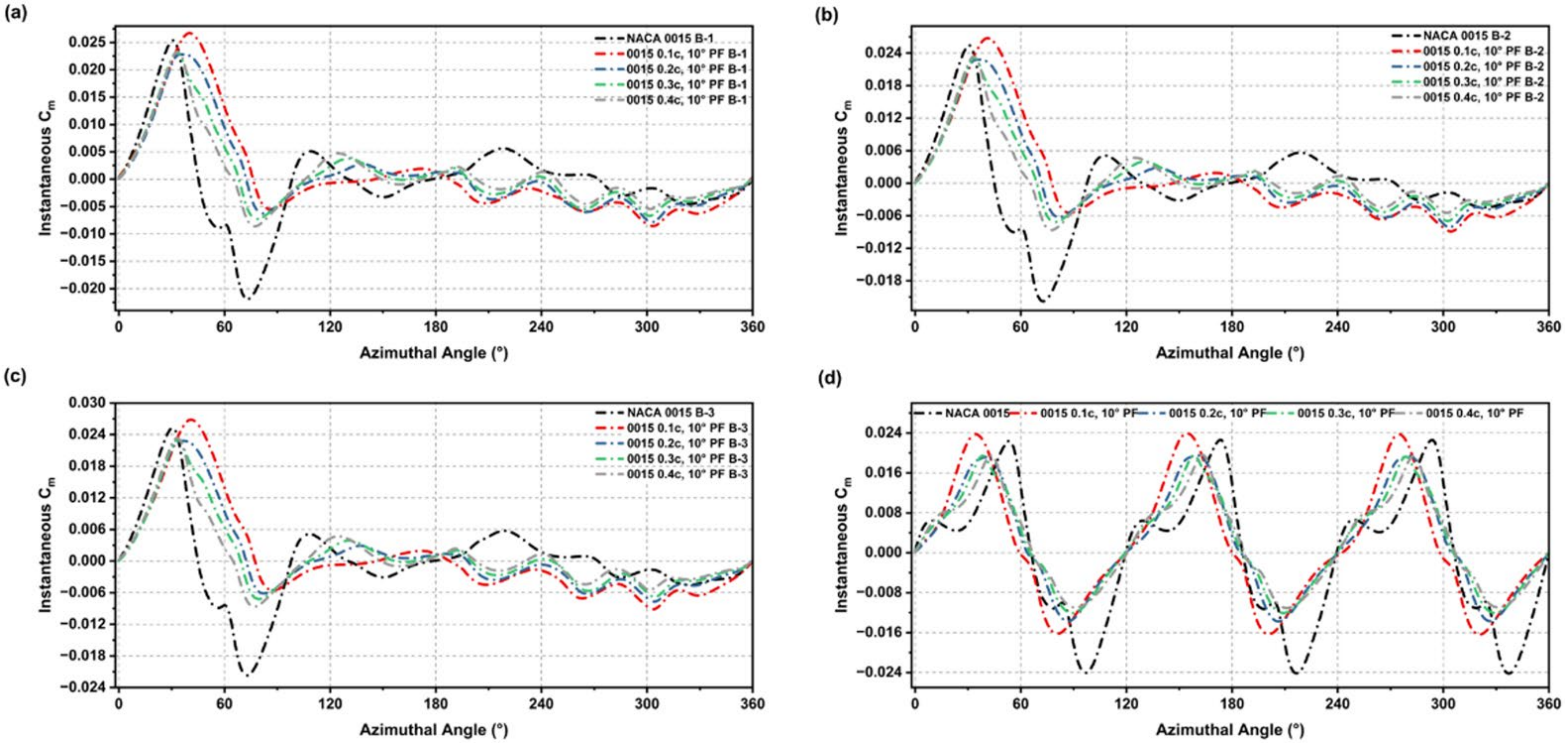
Instantaneous Cm for the full cycle of rotation, comparing NACA 0015 and PF configurations (0.1c to 0.4c) across different blade setups (B-1 To B-3) and the complete turbine.

**Fig. 10:**
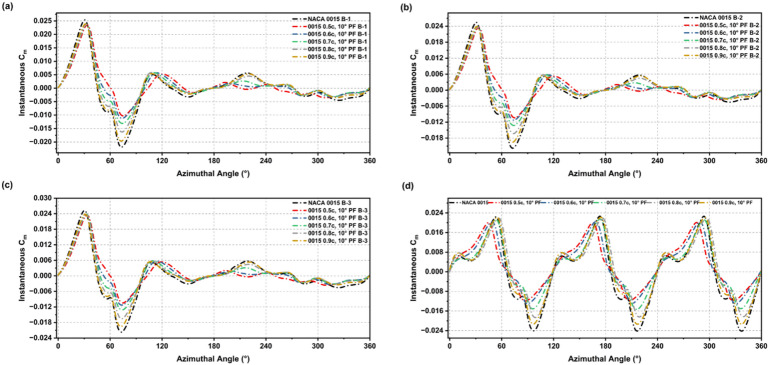
Instantaneous Cm for the full cycle of rotation, comparing NACA 0015 and PF configurations (0.5c to 0.9c) across different blade setups (B-1 To B-3) and the complete turbine.

**Fig. 11:**
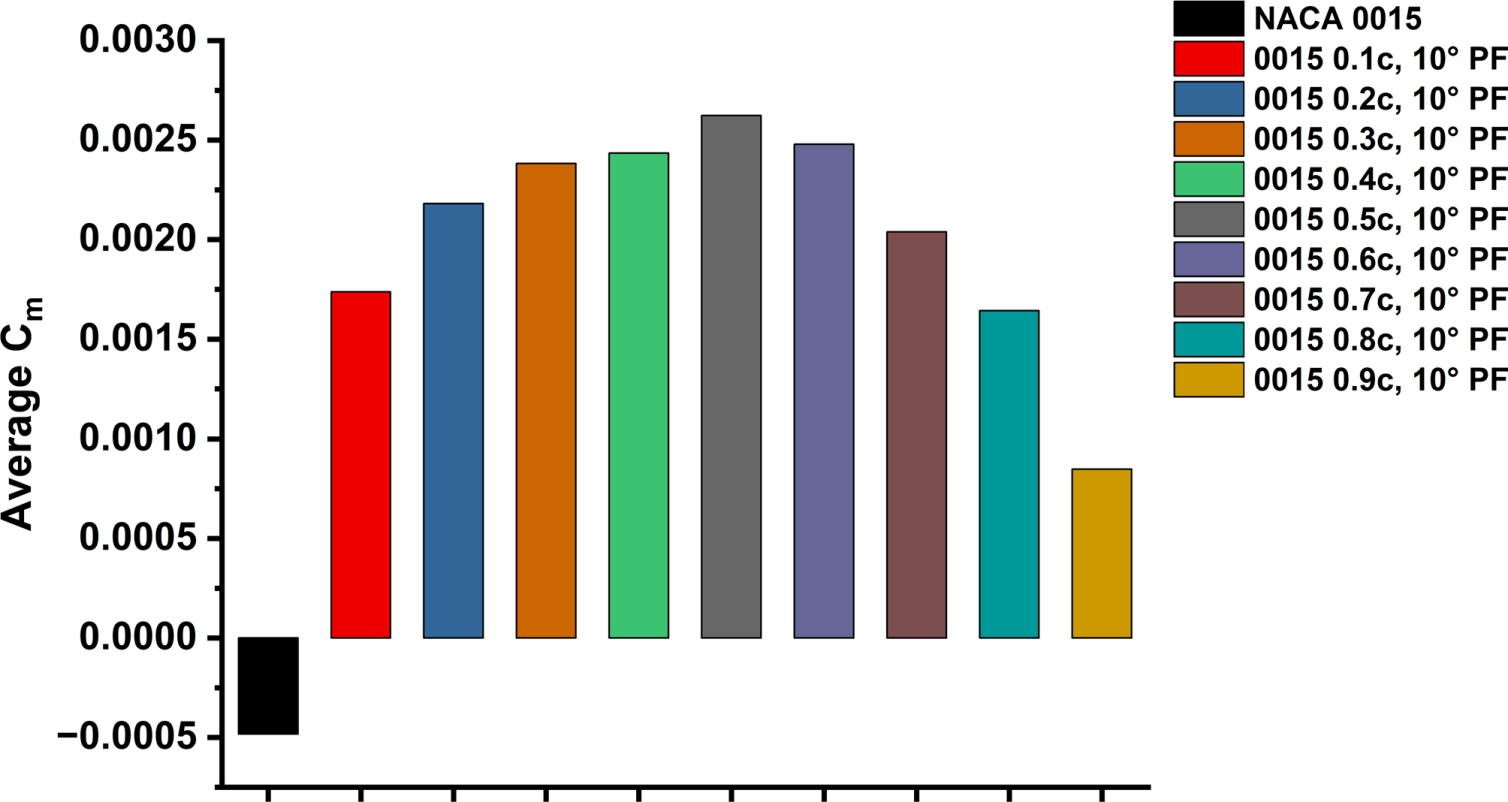
Average Cm for NACA 0015 aerofoil with PF configurations compared to the baseline aerofoil.

Torque performance was further examined under low-Re conditions (Re ≈ 5.4 × 10^4^, U_∞_ = 5 m/s, TSR = 0.8, and c = 0.16 m) to assess suitability for small-scale urban applications. As illustrated in Figs. 9, 10 and 11, all PF-modified configurations outperformed the baseline, which failed to produce positive average torque under these conditions. Mid-chord placements (0.3c–0.6c) proved most effective, with the 0.5c and 0.6c PFs maintaining consistent positive output across the azimuthal cycle while suppressing negative torque dips. In contrast, leading-edge (0.1c–0.2c) and trailing-edge (0.7c–0.9c) modifications, while boosting instantaneous peaks, induced deeper negative excursions due to adverse lift-drag trade-offs. Consequently, the 0.5c PF attained the highest average Cm, confirming it as the optimal configuration for enhancing start-up reliability and operational consistency in weak-wind environments.

### Performance of gurney flap

A GF is a small trailing-edge tab that increases camber, enhances lift, and delays separation through vortex generation. In Darrieus VAWTs, optimised GF placement improves starting torque and stability, as detailed in Table 2.

**Fig. 12:**
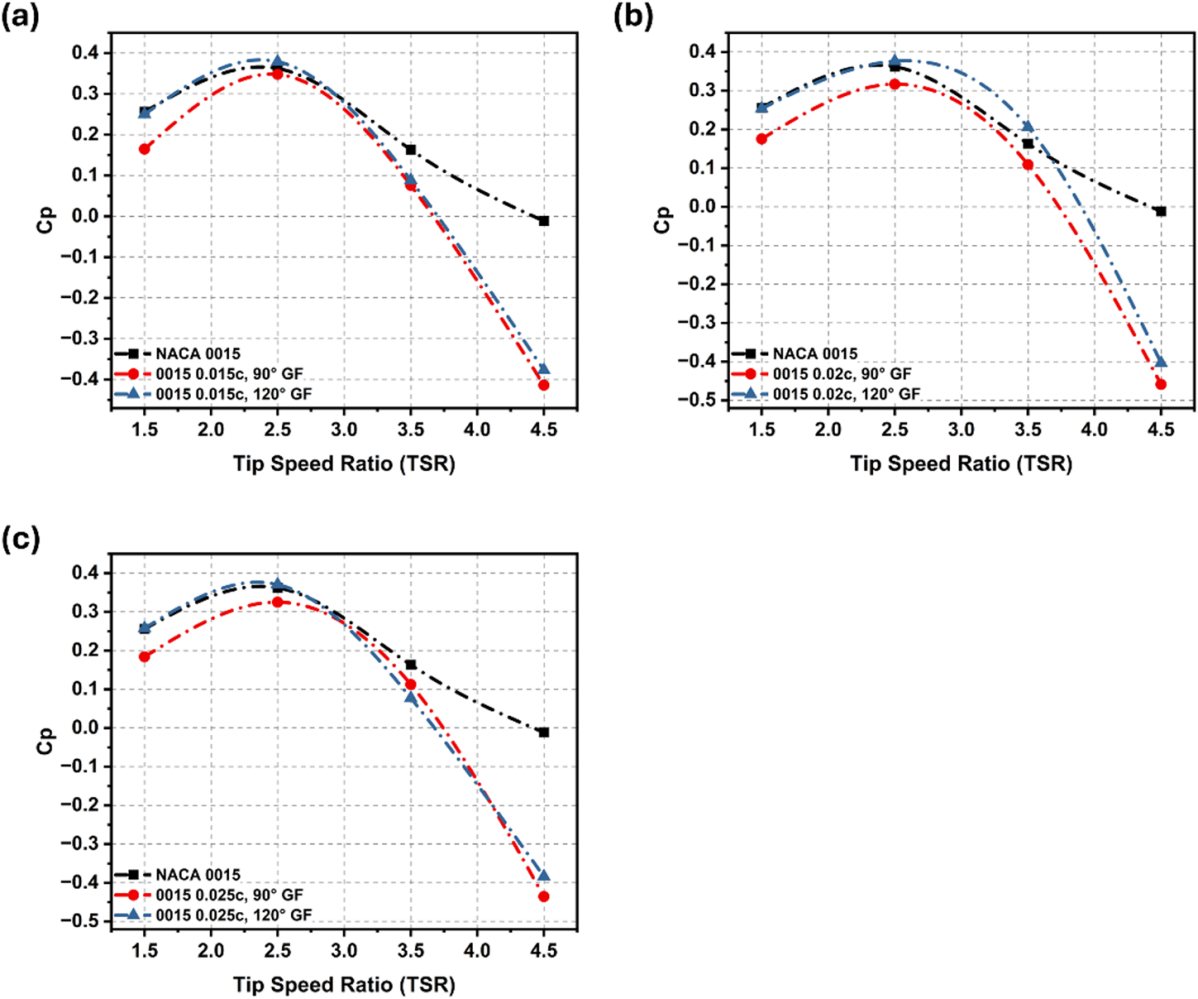
Cp comparison for flapless NACA 0015 aerofoil with GF configurations across various TSRs.

Figure 12a–c presents the effect of GF height and deflection angle on the Cp of the NACA 0015 aerofoil across TSR 1.5–4.5 under high-Re conditions. At TSR 1.5, 120° GFs with heights of 0.015c and 0.025c matched the baseline with Cp close to 0.25, whereas the 90° GFs showed noticeably reduced performance. At TSR = 2.5, the 120° GFs yielded optimal results, with the 0.015c configuration attaining Cp = 0.379, reflecting a 4.7% improvement over the baseline of 0.362. At TSR 3.5, only the 0.02c, 120° GF exhibited a distinct advantage, attaining a Cp of 0.206, which corresponds to a 26% improvement.

Most other cases gave negligible or negative gains. At TSR 4.5, none maintained positive Cp due to stall, though the 0.02c, 120° GF performed marginally better. Overall, GFs improved performance only at moderate TSRs, with 0.015c–0.025c at 120° proving most effective, while 90° deflections and extreme TSRs lowered efficiency.

**Fig. 13:**
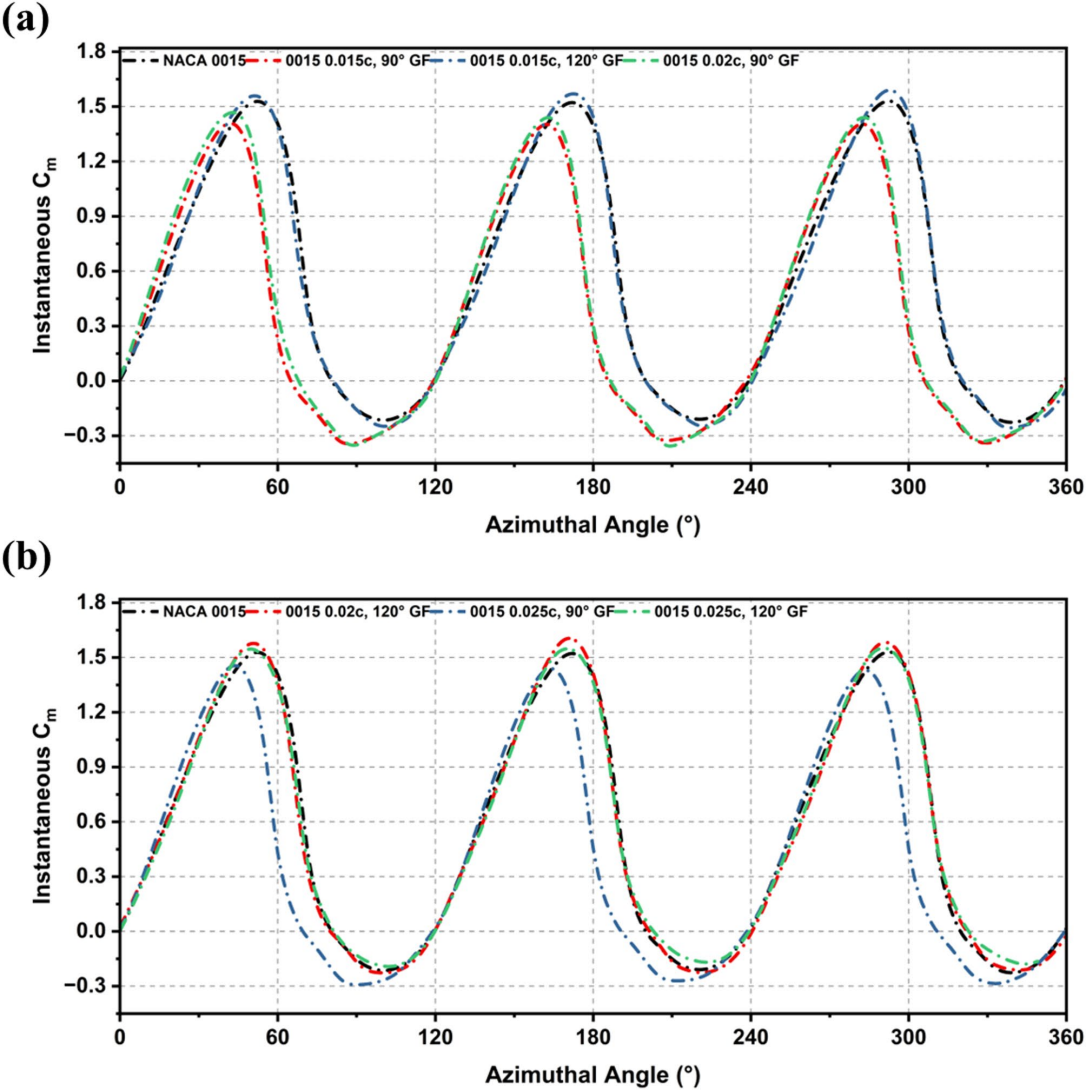
Instantaneous Cm variation over 0–360° rotation for NACA 0015 and its GF-modified configurations at TSR 1.5.

Figure 13 present the effect of GF height and deflection on the torque performance of NACA 0015 at TSR 1.5 and Re = 2 × 10^6^. The results are consistent with the Cp trends, with mid-sized 120° flaps enhancing performance, whereas smaller heights and 90° deflections offered limited benefit. The findings emphasise the strong dependence of torque performance on flap height and orientation. The smallest case (0.015c at 90°) delivered the poorest result, with an average Cm of 0.34, about 35% lower than the baseline (0.53). Increasing the deflection to 120° markedly improved performance, lifting Cm to 0.52 almost matching the baseline and reducing negative torque dips.

**Fig. 14:**
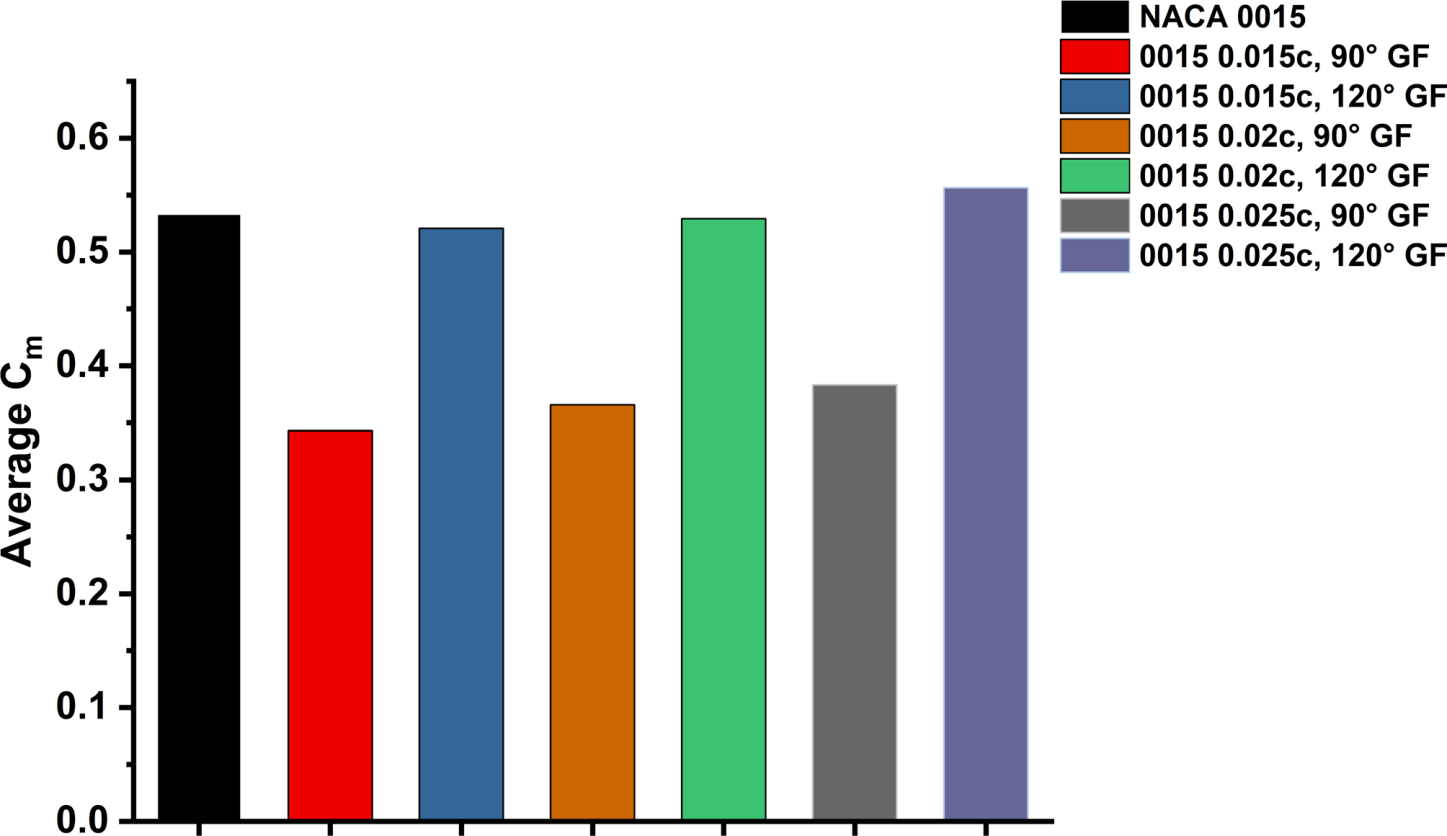
Comparison of average Cm for baseline NACA 0015 and GF-modified configurations, under TSR = 1.5.

Figure 14 shows that the advantage of a 120° deflection becomes more evident at larger flap heights. The 0.02c, 120° GF produced an average Cm of 0.53, matching the baseline, while the 0.025c, 120° GF yielded the highest torque at 0.56, a 6% improvement. In contrast, 90° GFs at similar heights were less effective, with Cm values of 0.36–0.42, indicating that perpendicular orientation suppresses lift and increases drag. In summary, the 0.02c and 0.025c GFs at 120° proved most effective, matching or surpassing the baseline Cm and improving torque stability. In contrast, 90° deflections consistently degraded performance, showing that moderate heights with swept-back orientation deliver clear torque benefits, while perpendicular GFs add little value.

**Fig. 15:**
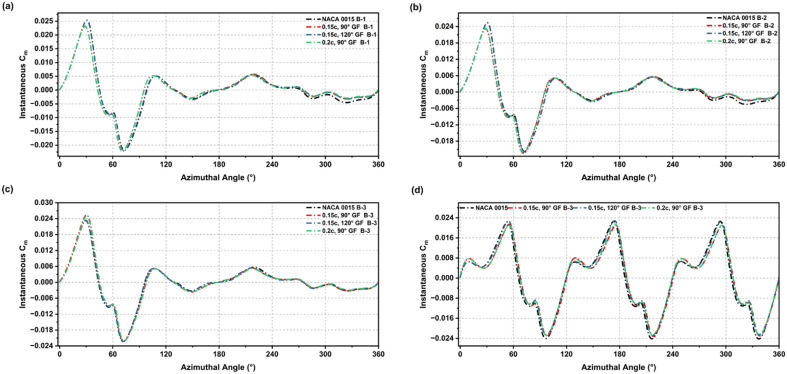
Instantaneous Cm across 360° rotation for NACA 0015 and GF-modified cases (0.015c, 90°; 0.015c, 120°; 0.02c, 90°) in blade setups B-1 to B-3 and the full turbine.

**Fig. 16:**
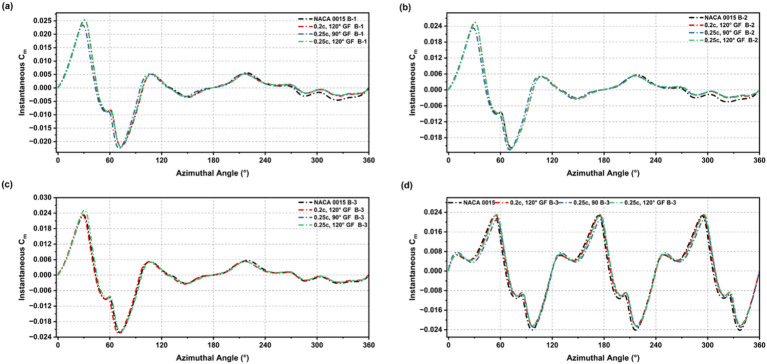
Instantaneous Cm across 360° rotation for NACA 0015 and GF-modified cases (0.02c, 120°; 0.025c, 90°; 0.025c, 120°) in blade setups B-1 to B-3 and the full turbine.

The incorporation of a GF on the NACA 0015 aerofoil resulted in distinct alterations of the instantaneous Cm behaviour at low-Re, highlighting significant aerodynamic modifications as depicted in Figs. 15 and 16. In all configurations, the baseline’s negative torque dips were reduced, and the transition to positive torque occurred earlier across each blade setup. GF-modified profiles showed smoother and more stable torque production over the full rotation cycle, demonstrating that GFs reduce the large negative fluctuations observed in the baseline aerofoil.

**Fig. 17:**
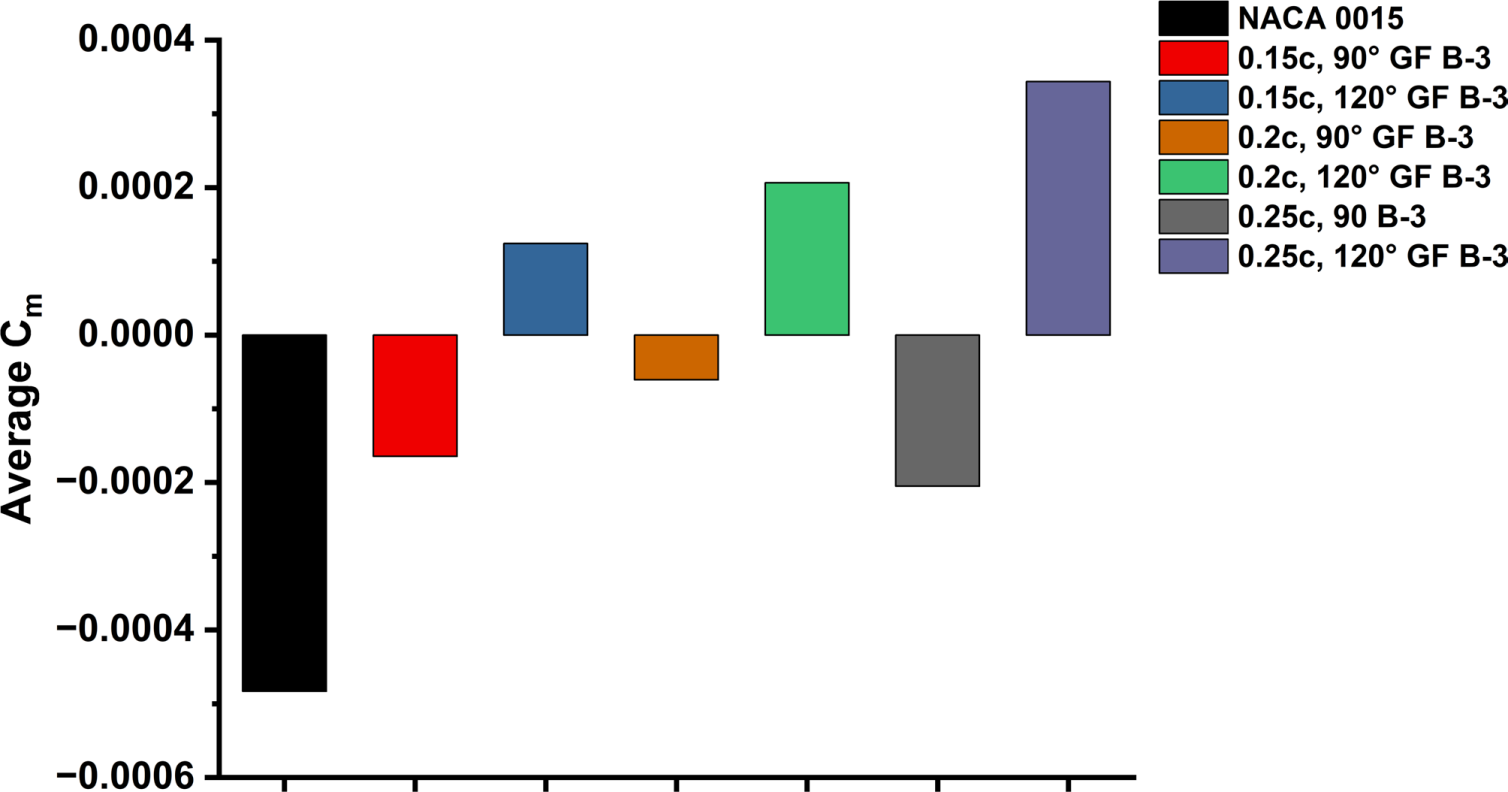
Average Cm of NACA 0015 and GF variants (0.015c, 90°-0.025c, 120°).

The improvement was most pronounced during the initial phase of rotation, as GF-modified blades produced earlier, steadier positive torque, leading to enhanced self-starting behaviour. Although the level of improvement varied depending on GF height and deflection angle, most configurations demonstrated overall better stability in torque output compared to the flapless aerofoil. Figure 17 summarises the influence of these modifications on the average Cm. GF-modified aerofoils exhibited higher average Cm than the baseline, confirming the positive effect of GF integration in enhancing starting torque and reducing negative contributions throughout the full cycle.

### Performance of hybrid plain gurney flap

After identifying the optimal PF and GF configurations, a hybrid PGF was developed by combining the stabilising influence of the PF with the lift-enhancing effect of the GF. The aim was to enhance energy capture, torque stability, and start-up capability across a range of TSRs. The PGF design was evaluated against both the baseline aerofoil and the best-performing PF configuration.

Two PGF configurations were considered: 0.015c GF at 120° with 0.5c, 10° PF (0.015 PGF) and 0.025c GF at 120° with 0.5c, 10° PF (0.025 PGF). The performance of the 0.5c, 10° PF and the baseline aerofoil were also evaluated for comparison. Their aerodynamic performance was analysed in terms of Cp, Cm, and torque ripple to assess self-starting behaviour, stall response, and efficiency at Re = 2 × 10^6^ with a wind speed of 10 m/s.

**Fig. 18:**
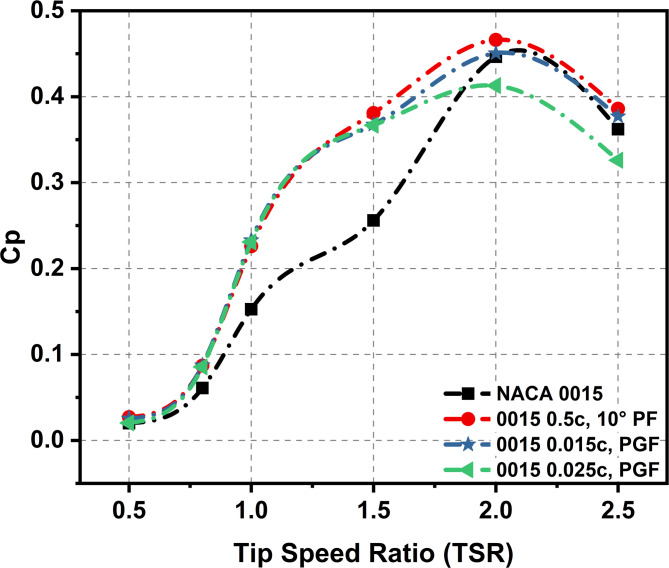
Cp comparison for NACA 0015 (flapless), 0.5c PF, and two PGF cases across TSRs 0.5–2.5 at Re = 2 × 10^6^.

Figure 18 present Cp trends for the flapless aerofoil, the 0.5c PF, and two PGF configurations (0.015c and 0.025c). At low TSRs (0.8–1.5), all modified cases achieved significant efficiency gains. At TSR = 0.8, Cp increased by 38% for the PF and the 0.015c PGF, while the 0.025c PGF achieved a slightly lower improvement of 35%. At TSR = 1, the 0.015c PGF recorded the highest gain of 52.7%, followed by the 0.025c PGF at 51% and the PF at 48%. At TSR = 1.5, all three configurations sustained strong performance, yielding improvements of about 49% for the PF and 43% for the PGFs.

At higher TSRs, performance differences became more pronounced. At TSR = 2, all cases reached peak Cp: the PF achieved 0.466, the 0.015c PGF reached 0.451 with only a marginal gain, while the 0.025c PGF declined by 7.5% relative to the flapless. For TSR 2.5, the PF case sustained a modest improvement of 6.6%, the 0.015c PGF delivered a limited gain of 4.1%, and the 0.025c PGF underperformed, showing a 10% reduction compared with the baseline.

**Fig. 19:**
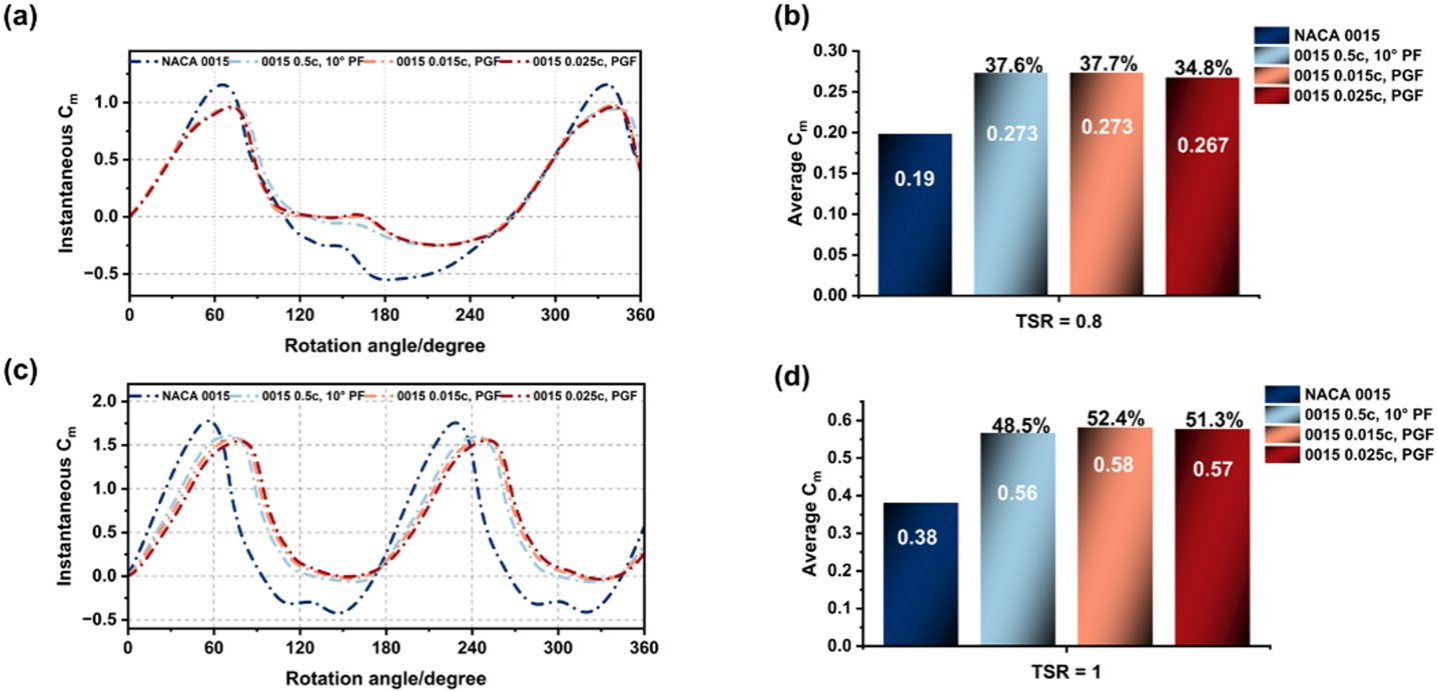
Instantaneous and average Cm profiles of NACA 0015 baseline, PF, and PGF at TSR = 0.8 and 1.0.

**Fig. 20:**
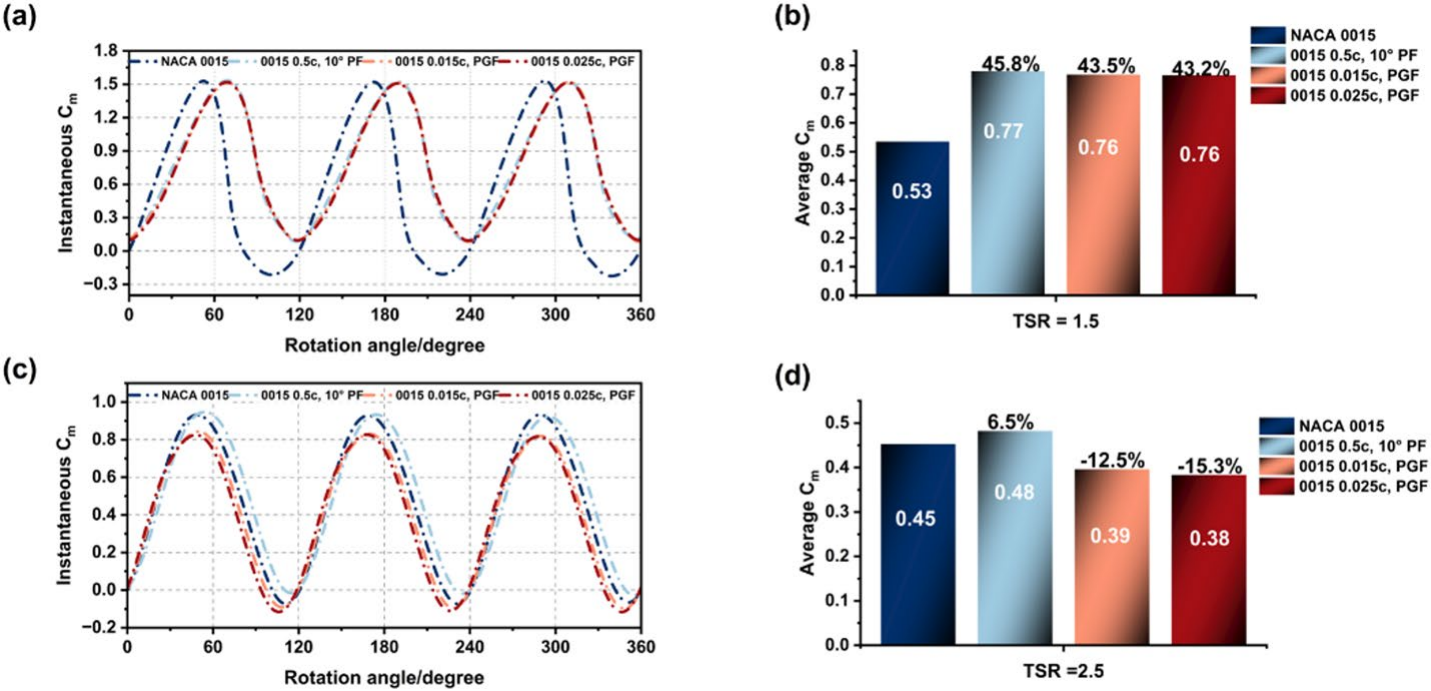
Instantaneous and average Cm profiles of NACA 0015 baseline, PF, and PGF at TSR = 1.5 and 2.5.

The instantaneous and average Cm characteristics of the baseline, PF, and PGF configurations were analysed across TSR 0.8–2.5 (Figs. 19 and 20). At start-up and low speeds (TSR 0.8–1.0), trailing-edge modifications significantly enhanced performance. Both the PF and 0.015c PGF increased average Cm by approximately 44% at TSR 0.8 compared to the baseline (0.19 to 0.273). At TSR 1.0, the PGF demonstrated a slight advantage (Cm = 0.58) over the PF (0.56), attributed to higher lift generation.

However, as rotational speed increased, the aerodynamic behaviour diverged. At TSR 1.5, improvements persisted but the performance gap narrowed. By TSR 2.5, the PGF configurations suffered a notable decline, dropping average Cm to 0.38–0.39 (below the baseline of 0.45) due to increased form drag and weaker flow attachment. In contrast, the PF maintained aerodynamic stability with a marginal gain (0.48). This confirms that while PGFs excel in the start-up regime, the PF configuration offers superior consistency across the full operational range.

**Fig. 21:**
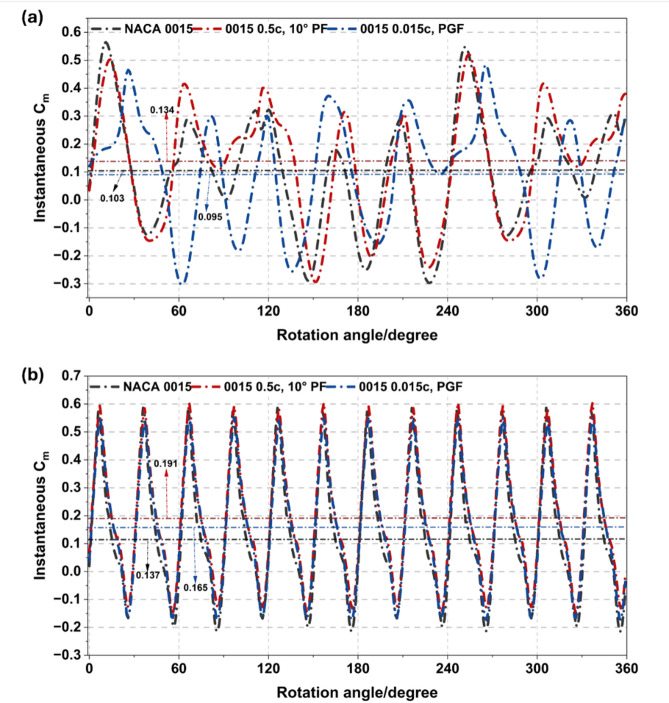
Instantaneous Cm profiles of NACA 0015 baseline, PF, and PGF at TSR = 0.2 and 0.5.

The influence of flap modifications on low-speed starting torque was evaluated at the initial start-up phase (TSR = 0.2) and early acceleration (TSR = 0.5), as shown in Fig. 21. At TSR = 0.2, the 0.5c, 10° PF significantly enhanced torque stability, increasing mean Cm by 29.8% over the baseline (0.103–0.134) by delaying flow separation and maintaining attached flow during the upwind cycle. In contrast, the 0.015c PGF suffered a 7.9% performance reduction (Cm = 0.095) as stronger trailing-edge vortices increased downwind negative torque.

As the turbine transitioned to TSR = 0.5, both configurations improved, but the PF maintained its dominance. The PF increased mean Cm to 0.192 (a 39.7% gain over the baseline), sustaining a smooth positive torque profile across the azimuthal cycle. While the PGF performance recovered (Cm rising to 0.166) due to stabilized wake circulation, the PF consistently delivered superior start-up reliability and smoother load progression, confirming it as the optimal passive device for low-speed operation.

**Fig. 22:**
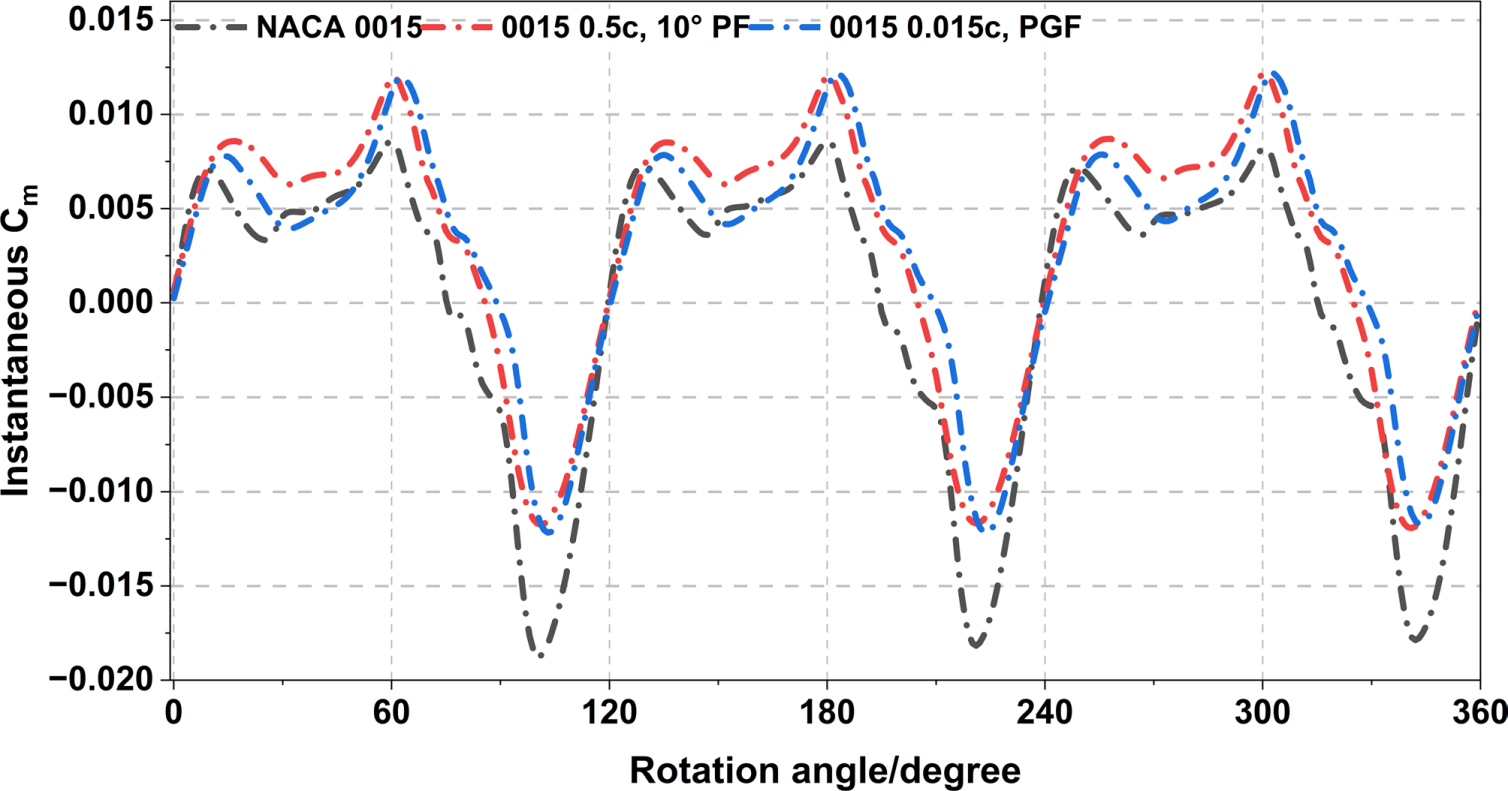
Instantaneous Cm profiles of NACA 0015 baseline, PF, and PGF at TSR = 0.5 and Re = 6.0 × 10^4^.

Figure 22 shows the instantaneous Cm variation with rotation angle for the NACA 0015 aerofoil and two modified configurations at a TSR of 0.5 and a Re of about 6.0 × 10^4^. The baseline aerofoil produces clear periodic torque fluctuations with alternating positive and negative values, reflecting the cyclic change in aerodynamic loading as each blade rotates. Adding a 0.5c, 10° PF and a 0.015c PGF increases the positive Cm peaks and slightly reduces the negative troughs. The PF configuration shows the highest torque amplitude, indicating stronger lift enhancement and better flow attachment near the trailing edge. Both modifications enhance the average torque and reduce the magnitude of negative torque, leading to smoother torque variation and improved self-starting behaviour under low-Re conditions.

In contrast Figs. 21 and 22 compare the 2.5 m (Re ≈ 2.7 × 10^5^) and 1 m (Re ≈ 6.0 × 10^4^) rotors, showing that Re strongly affects flap performance. In Fig. 21, the 0.5c, 10° PF achieves the highest improvement in mean Cm, increasing by up to 39.7%, while the PGF provides smaller and less stable gains. In Fig. 22, both flaps still enhance positive torque under low-Re conditions but with reduced effectiveness due to laminar separation. Overall, the PF remains the most effective and stable configuration across both scales, while larger high-Re turbines require smaller flap deflections or chord ratios to maintain efficiency and minimise drag.

Table 5 summarizes the aerodynamic performance (Cp) of the baseline, 0.5c 10° PF, and 0.015c PGF configurations. The analysis identifies distinct operational regimes:

1. Low-speed regime (TSR 0.2–0.5): The 0.5c PF demonstrated superior start-up capability, improving mean Cm by 29.8–39.8% and Cp by nearly 40% relative to the baseline. By maintaining attached flow throughout the upwind cycle, the PF provides the most reliable torque response for self-starting in weak winds.
2. Transition regimen (TSR 0.8–1.5): Both configurations delivered their peak aerodynamic benefits, achieving substantial Cp improvements of 38–53%. The PGF exhibited a slight advantage at TSR 1.0 (+ 52.7% vs. +48.6% for PF) due to enhanced Gurney-induced lift, indicating its potential for moderate wind conditions.
3. Rated-speed regime (TSR 2.0–2.5): Performance trends diverged as rotational speed increased. The PF maintained its advantage, improving Cp by 4.3–6.5% over the baseline, while the PGF’s relative gain diminished significantly due to increased form drag.

Overall, the 0.5c, 10° PF emerged as the optimal configuration for practical application. It balances high start-up torque with sustained efficiency at rated speeds, offering a more stable aerodynamic profile than the PGF, which is structurally more complex and drag-sensitive at higher TSRs.

**Table 5:** Summary of aerodynamic performance for baseline and modified configurations.

TSR	NACA 0015 (Cp)	0.5c, 10° PF (Cp)	0.015c, PGF (Cp)
0.2	0.006	0.0085 (29.8)	0.006 (− 7.8)
0.5	0.021	0.03 (39.8)	0.0265 (21%)
0.8	0.063	0.087 (38.7%)	0.087 (38.7%)
1.0	0.152	0.226 (48.6%)	0.233 (52.7%)
1.5	0.255	0.381 (49.1%)	0.367 (43.6%)
2.0	0.446	0.466 (4.3%)	0.450 (0.9%)
2.5	0.362	0.386 (6.5%)	0.377 (4.1%)

The aerodynamic mechanisms governing the flap configurations can be explained through their influence on flow development around the blade. For the PF, the 10° mid-chord deflection increases the effective camber and shifts the zero-lift angle, generating a stronger suction peak on the upper surface and delaying separation. This improves lift and enhances torque at low TSR^48,49^.

The GF acts differently from the PF. The trailing-edge tab generates a pair of counter-rotating vortices in the wake that strengthen circulation and improve pressure recovery, thereby enhancing lift but increasing form drag. These near-wake flow structures and their lift-drag trade-off have been experimentally demonstrated by Filippone and Brown and comprehensively reviewed by Wang et al.^50,51^. In the present study, a 120° trailing-edge tab angle was adopted to replicate the perpendicular GF configuration and evaluate its effect under low-Re conditions. At higher TSR, intensified vortex shedding increases form drag and reduces aerodynamic efficiency.

The PGF configuration combines these effects. The PF increases camber and lift, while the GF stabilises the wake and delays separation, resulting in higher average torque and smoother flow at low TSR^29,52^. However, as TSR increases, trailing-edge vortex interactions become stronger and more unsteady, causing wake thickening and increased pressure drag^51^. The relative inflow angle decreases, and vortex shedding becomes dominant, leading to greater energy loss and diminished aerodynamic benefit. Experimental and numerical studies on VAWTs fitted with GFs have reported similar behaviour, showing improved performance at low TSR but a decline in power coefficient at higher TSR due to drag growth and wake instability^27,28,53^.

Based on the results in Table 5, the aerodynamic advantage of the PGF configuration declines beyond TSR = 2.0, where the drag induced by the Gurney element outweighs its lift contribution, leading to reduced efficiency compared with the PF. In contrast, the 0.5c, 10° PF maintains consistent improvement across the full TSR range, sustaining higher Cp values and smoother torque characteristics under increasing rotational speeds. These trends indicate that the aerodynamic performance of trailing-edge modifications in Darrieus VAWTs is primarily driven by their influence on effective camber, circulation, and wake stability. The trade-off between lift enhancement and drag increase defines the most suitable flap geometry and operating TSR, with the PF configuration achieving the most balanced and stable performance.

### Static torque analysis from initial 0° to 75° rotation angles

**Fig. 23:**
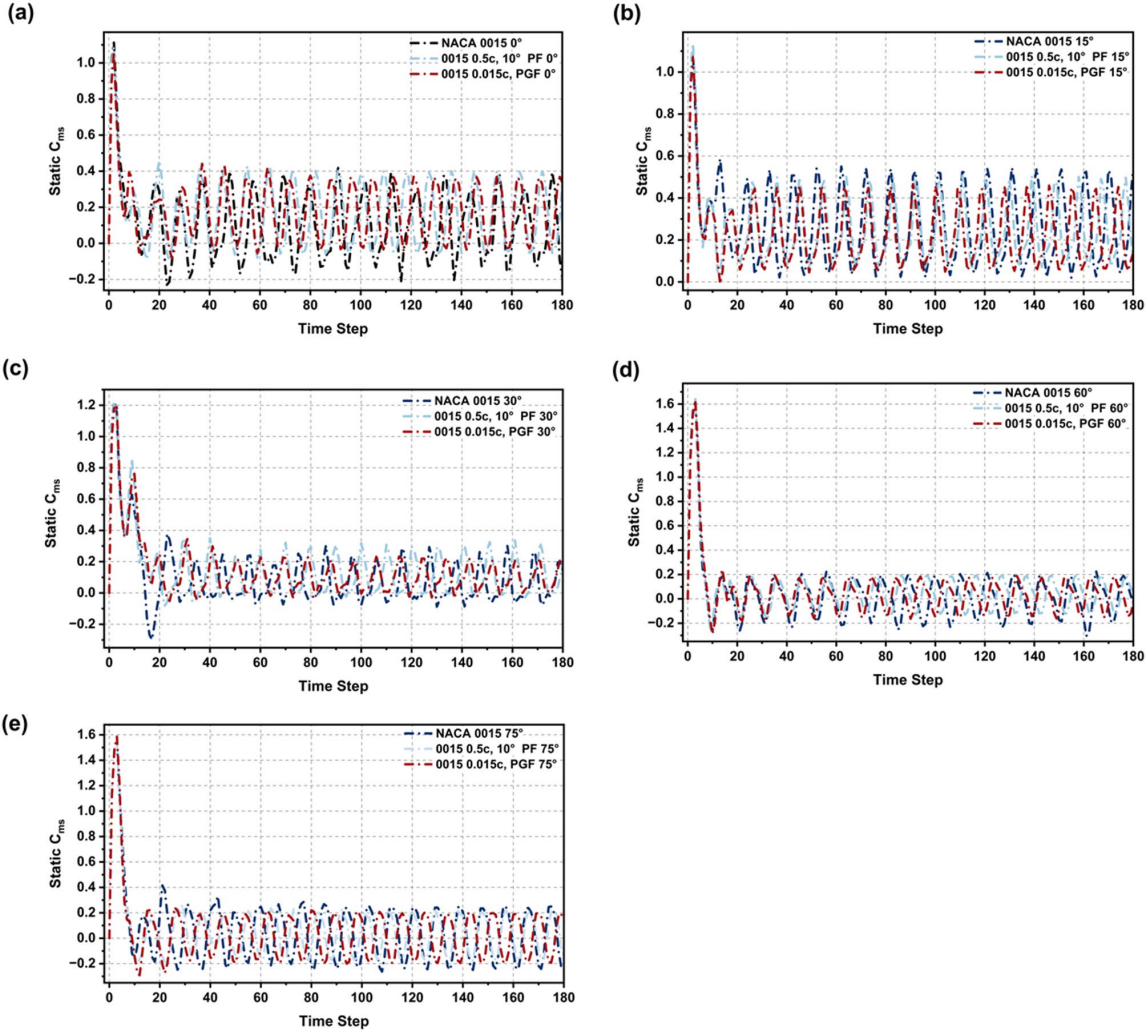
Static torque coefficient (Cm) variation over 180-time steps for flapless NACA 0015, 0.5c PF, and 0.015c PGF across rotation angles (0° to 75°).

The analysis of Fig. 23; Table 6 evaluates the starting torque performance of NACA 0015, 0.5c 10° PF, and 0.015c PGF under Re = 2 × 10^6^ conditions. At 0° rotation, both PF and PGF outperform the baseline, with PGF achieving a 66% and PF a 55% improvement in average static Cm. These modifications also reduce torque fluctuations, improving early rotation stability.

At 15° and 75°, the baseline NACA 0015 shows better performance, indicating its stability at critical angles where the modified profiles experience torque reduction. At mid-angles (30° and 60°), the 0.5c, 10° PF consistently delivers higher Cm values and smoother trends, while 0.015c PGF offers comparable improvement at 30°. Both modifications reduce negative torque dips and enhance aerodynamic efficiency.

In summary, the 0.015c PGF is recommended for starting torque applications, while the 0.5c, 10° PF is the optimal choice for improving performance across most mid-rotational angles. For operations requiring stability at specific critical angles, the baseline NACA 0015 remains the preferred option. The final selection of configuration should depend on the operational priorities and the specific torque requirements of the turbine system.

**Table 6:** Average cm comparison of baseline vs. PF and PGF configurations at various rotation angles (0° to 75°).

Rotation angle (°)	NACA 0015 (Cm)	0.5c, 10° PF (Cm)	0.015 PGF (Cm)
0°	0.109	0.170 (55%)	0.181 (66%)
15°	0.268	0.257 (− 4.1)	0.23 (− 14%)
30°	0.091	0.144 (59%)	0.139 (53%)
60°	0.039	0.07 (76%)	0.059 (49%)
75°	0.067	0.064 (− 4.4%)	0.051 (− 24%)

### Analysis of velocity, pressure, and streamline contours

**Fig. 24:**
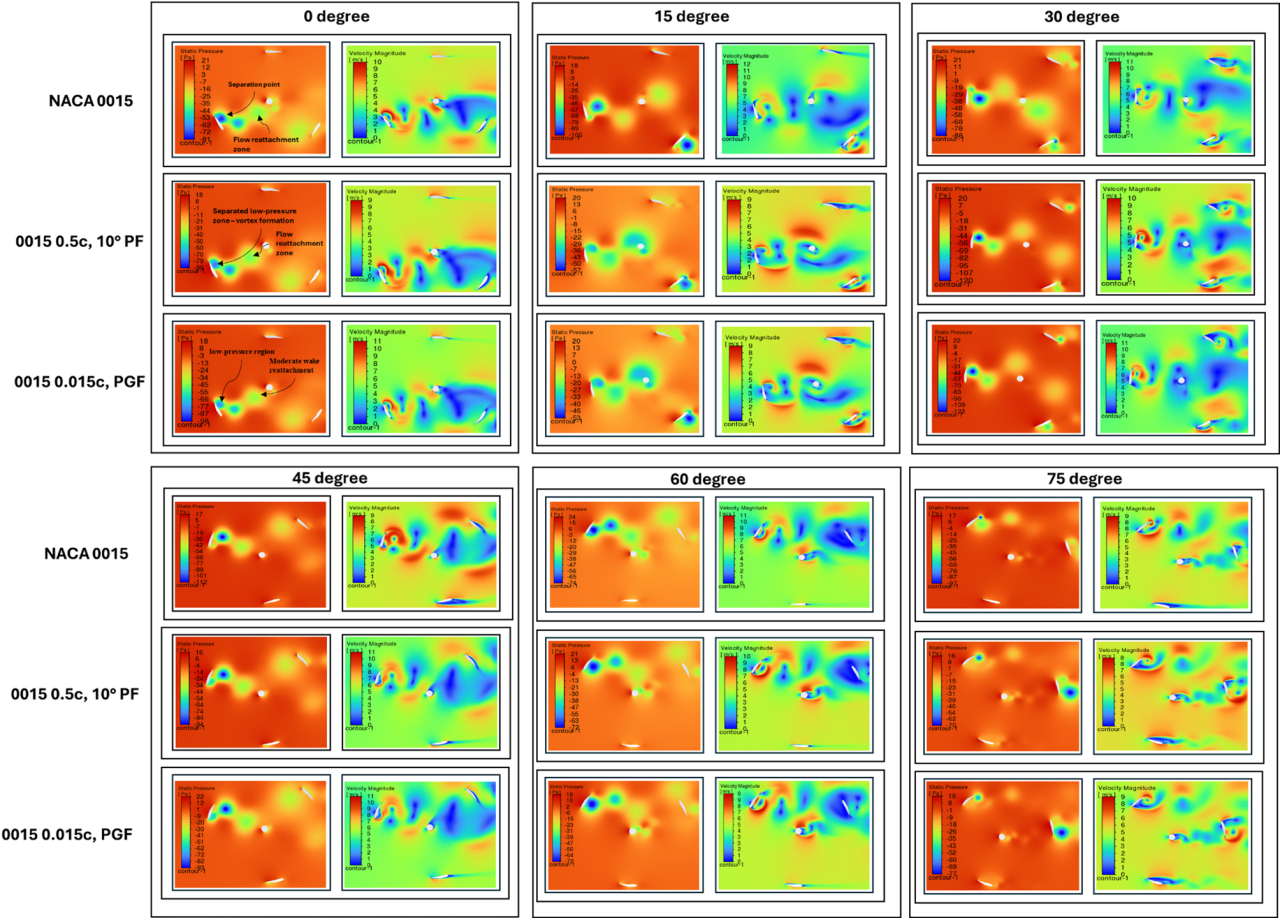
Velocity and pressure fields at high Re TSR = 1.

The analysis of pressure and velocity contours in Fig. 24 under Re = 2 × 10^6^ conditions highlight the aerodynamic behaviour of the flapless aerofoil, 0.5c PF, and 0.015c PGF configurations. At 0°, the baseline NACA 0015 shows a broad low-pressure region extending downstream, indicating early separation and poor pressure recovery. The 0.5c, 10° PF displays a confined low-pressure zone near the trailing edge, signifying controlled separation and partial reattachment that stabilises the wake and enhances initial torque. The 0.015c PGF produces a stronger but more localised low-pressure region, increasing suction and circulation while maintaining moderate wake reattachment.

At 15°, the baseline exhibits early detachment and weak recovery, confirming strong separation and low efficiency. The 0.5c, 10° PF maintains stable suction with a confined low-pressure region and partial reattachment, reducing wake losses. The 0.015c PGF forms a concentrated trailing-edge suction zone with smoother reattachment and symmetric wake development, improving overall flow stability and pressure recovery.

At 30°-45°, the baseline experiences progressive separation and an unsteady wake dominated by vortex shedding. The 0.5c, 10° PF delays separation toward the trailing edge, maintaining partial reattachment and improved wake organisation. The 0.015c PGF enhances circulation and suppresses large-scale vortices, yielding smoother suction distribution and a more coherent wake, leading to better lift and stability than PF and baseline cases.

At 60°-75°, the baseline shows complete flow detachment and strong vortex interaction, typical of full stall. The 0.5c, 10° PF exhibits moderate trailing-edge separation but retains partial mid-chord attachment, narrowing the wake and improving recovery. The 0.015c PGF maintains smaller low-pressure vortices, symmetric wake formation, and early reattachment, confirming superior flow control, stall resistance, and aerodynamic efficiency.

**Fig. 25:**
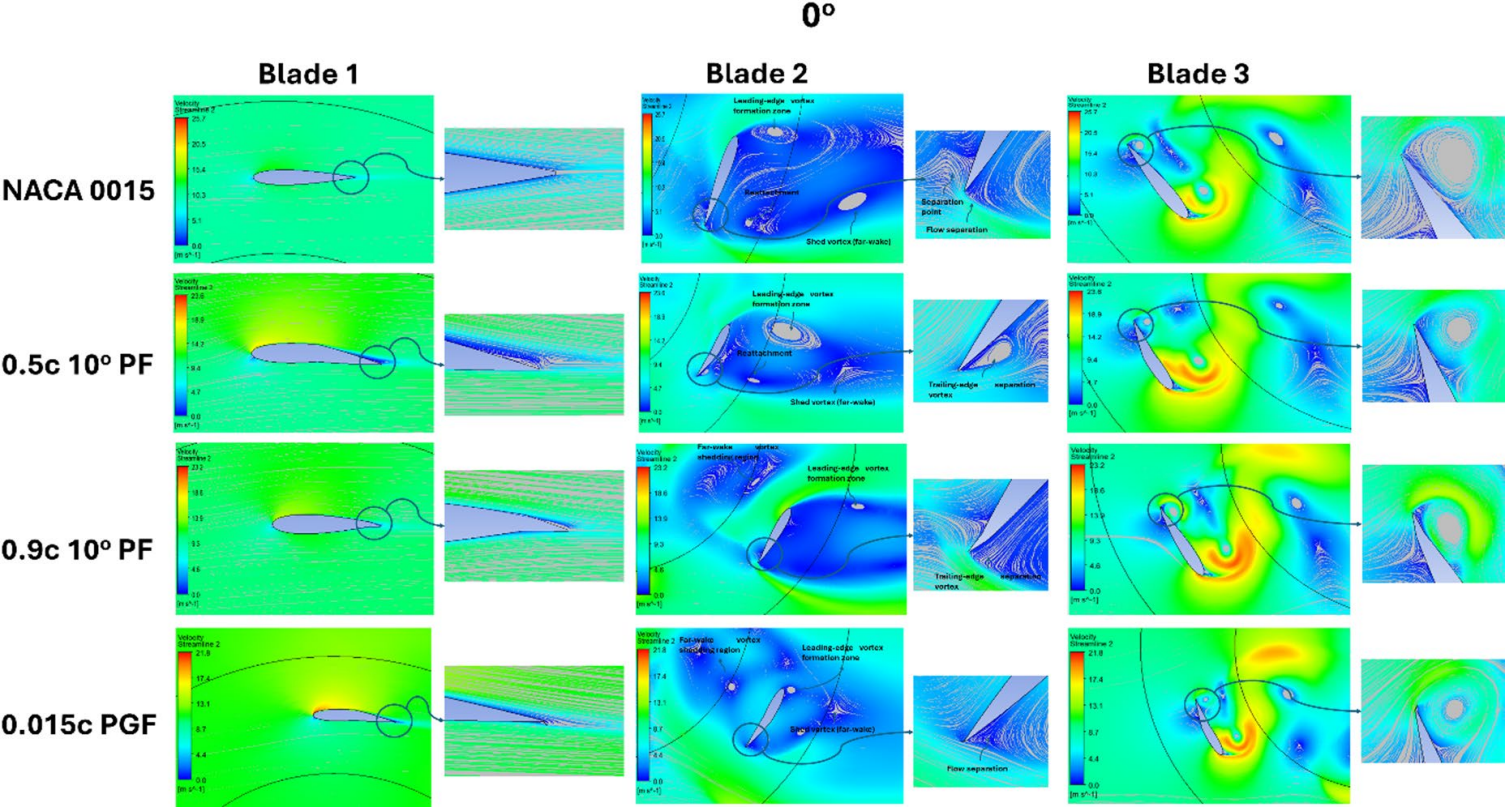
Instantaneous velocity-streamline fields at 0° for NACA 0015 and modified blades (0.5c, 10° PF, 0.9c, 10° PF, 0.015c PGF).

Figure 25 shows the velocity-streamline fields at 0° for all configurations. In NACA 0015, Blade-1 separates near the leading edge (no reattachment), while Blades-2-3 shed large TE vortices and form a broad wake. With 0.5c, 10° PF, Blades-1-2 exhibit delayed separation with partial reattachment; TEV intensity and wake deficit reduce. The 0.9c, 10° PF maintains attached flow with a distinct reattachment before the trailing edge and the weakest shedding across all blades. The 0.015c PGF increases suction near the TE and forms a small counter-rotating pair, improving lift but leaving slightly stronger wake persistence than 0.9c PF.

### Comparative performance evaluation

Table 7 compares the aerodynamic performance of the present trailing-edge modifications with previously published studies on Darrieus-type VAWTs.

The PF configuration achieved the highest efficiency, with a 49.1% increase in Cp at TSR 1.5 and a peak Cp of 0.466, showing a strong improvement in lift and torque stability. The optimal PGF, formed by a 0.5c, 10° PF combined with a 0.015c GF at 120°, recorded the largest performance gain of 52.7% at TSR 1.0 and exhibited the most uniform self-starting torque. The GF alone produced smaller gains of + 4.7% at TSR 2.5 (0.015c) and + 26% at TSR 3.5 (0.02c), indicating a useful contribution only at higher TSR values.

In contrast, earlier investigations by Zhang et al. (2021), Chen et al. (2020), Kord and Bazargan (2024), and Chen et al. (2023) reported Cp gains of 5–14% under comparable conditions^17,29,54,55^. This clear improvement demonstrates that the proposed PF and PGF configurations extend the aerodynamic capability of Darrieus VAWTs, especially in low-Re environments typical of urban and small-scale applications.

**Table 7:** Comparison of Cp improvements from the present trailing-edge modifications with previously published Darrieus-type VAWT studies.

Study	Modification	TSR range	Maximum verified Cp improvement	Key remarks
Present study	PF (0.5 c, 10°), PGF (0.015c), GF (0.015c, 0.02c @ 120°)	0.2–4.5	PF + 49.1% (λ = 1.5, Cp = 0.466); PGF + 52.7% (λ = 1.0, Cp = 0.233); GF + 4.7% (λ = 2.5, 0.015c), + 26% (λ = 3.5, 0.02 c)	PF: highest efficiency and stable torque from low to high TSR PGF: best self-start and low-TSR performance, reduced efficiency beyond TSR 2.0
Zhang et al.^17^	Split flap (10° deflection)	1.0–3.0	+ 7.7% (at optimum λ)	Improved low-TSR lift; slight drag increase above λ = 2
Chen et al.^54^	Active switching Gurney flap	0.8- 2.0	+ 5.1% (at maximum power)	Active control stabilises flow; small efficiency gain
Kord and Bazargan^55^	Inboard Gurney flap (0.75%c) on J-shaped Darrieus VAWT	0.5–2.25	+ 12.35% (at TSR 2.25)	Inboard GF delays stall by 14° and boosts power at high TSR
Chen et al.^29^	PGF (1.5%c) and SGF (6%c) on NACA 0021	1.6–3.3	PGF + 6.4% (Cp_max_ = 0.399); SGF + 13.9% (at TSR = 2.62)	SGF reduces drag and improves lift/drag ratio; PGF better at low TSR, SGF at high TSR

### Design and fabrication of the turbine

The Darrieus VAWT was designed with a chord length of 0.15 m, a blade height of 1 m, and a rotor diameter of 1 m, dimensions chosen for low wind speed and rural applications. manufacturing process for the Darrieus VAWT blade was carried out at the KLEF Fab Lab using PETG filament and 3D printing to ensure accuracy, strength, and surface quality.

Each blade consisted of six 0.167 m segments. Printing parameters were defined through G-code simulation, and a lattice infill was applied to optimise weight and strength. Mounting holes were integrated during printing to simplify assembly. The full blade weighed about 600 g, giving 1.8 kg for three blades, with 2 kg of filament required after accounting for supports and misprints. At ₹1400/kg, the total material cost was approximately ₹4200 (≈ $50 USD).

**Fig. 26:**
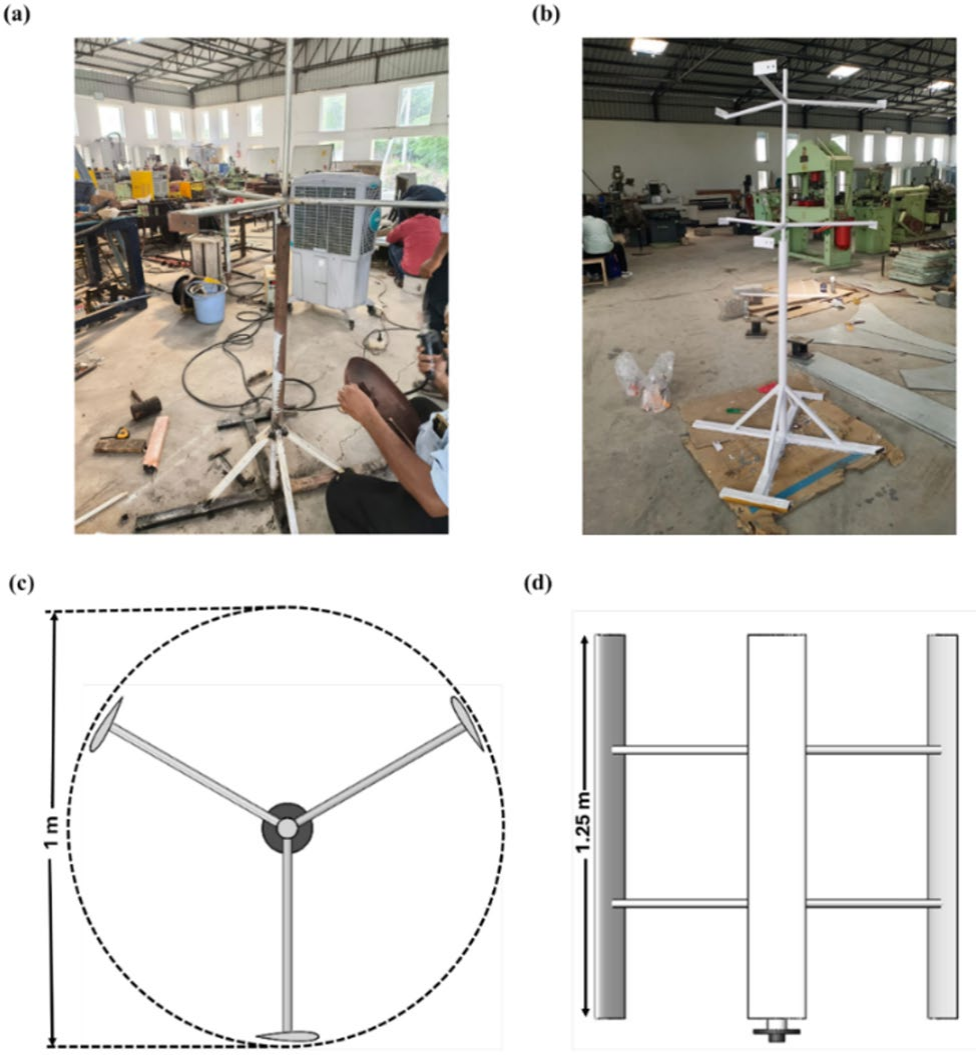
(**a**) Frame fabrication, (**b**) Assembled structure, (**c**) Top view of the 3D model, (**d**) Side view of the Darrieus wind turbine.

The turbine assembly included a mild steel frame, stable base, precision bearings, and interchangeable mounts for baseline and modified blades. Figure 26a,b show fabrication and assembly, while Fig. 26c,d present 3D model views. This process provided a cost-effective and repeatable method for prototype development.

**Fig. 27:**
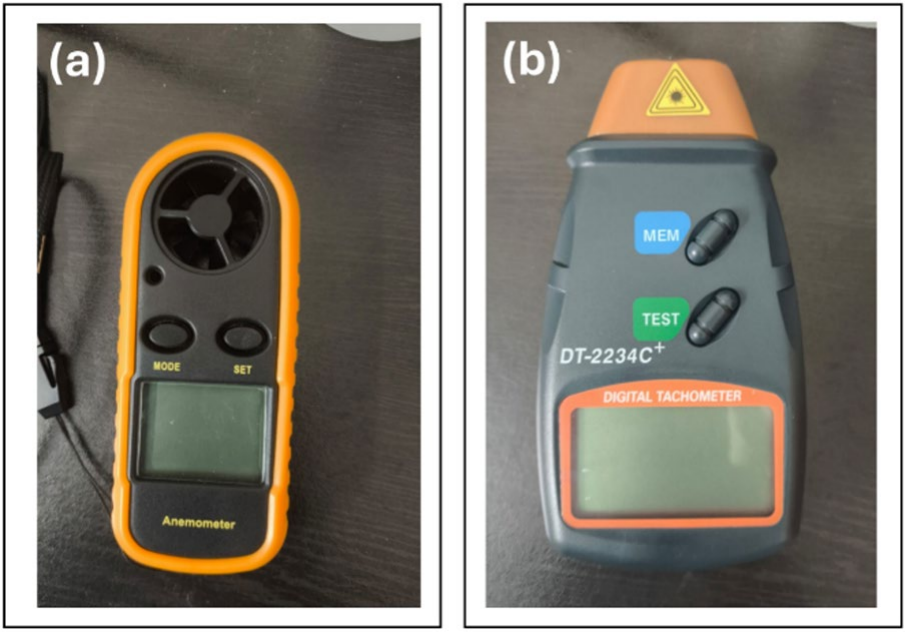
Instruments used for VAWT prototype testing: (**a**) Digital anemometer for wind speed, (**b**) DT-2234 C+ tachometer for rotor RPM.

Figure 27 illustrates the instrumentation employed for real-time data acquisition during the experimental validation of the Darrieus VAWT prototype. The anemometer (Fig. 27a) provided point measurements of incident wind velocity, critical for correlating environmental input with turbine performance. The digital tachometer (Fig. 27b) enabled precise measurement of shaft speed through laser-based detection, offering non-intrusive and accurate monitoring of rotor dynamics.

Wind uniformity during outdoor testing was maintained by continuously monitoring wind velocity with a digital anemometer positioned 0.5 m upstream of the turbine at hub height. Measurements were taken only under steady wind conditions, with velocity fluctuations restricted to ± 0.2 m/s around the mean for at least 60 s. Each test was repeated three times, and the averaged RPM values were used for performance evaluation. These procedures ensured consistent inflow conditions and reduced the influence of short-term turbulence and gusts on the recorded data.

The prototype was mounted on low-friction bearings to minimise mechanical resistance and ensure free rotation. Prior to testing, alignment was verified, and empty-spin trials confirmed negligible frictional torque. Wind velocity was measured using a digital anemometer (accuracy ± 0.1 m/s), and shaft rotational speed was recorded with a contactless optical tachometer (accuracy ± 1.5%). Each test was repeated three times, and the variation in recorded RPM remained within ± 2%, confirming data consistency and reliability. Considering both the instrument accuracy and repeatability variation, the overall uncertainty in RPM measurements was estimated at approximately ± 2.5%, which is acceptable for small-scale prototype testing.

### Experimental results

**Fig. 28:**
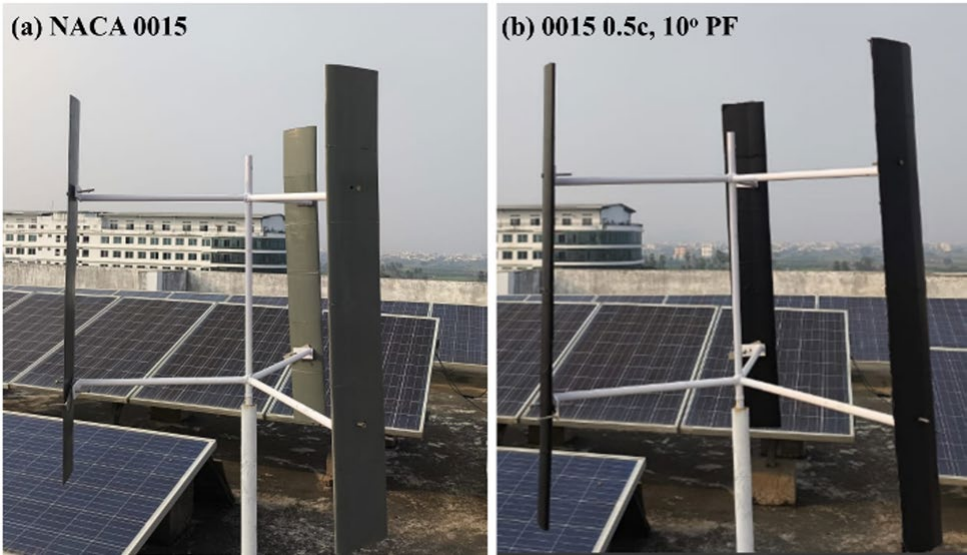
Darrieus VAWT prototype during testing with (**a**) baseline NACA 0015 blades and (**b**) blades modified with a 0.5c, 10° PF.

The experimental turbine was evaluated in two configurations: the baseline with NACA 0015 blades (Fig. 28a) and the modified design with the optimised 0.5c, 10° PF blades (Fig. 28b). This setup was used to examine the relative improvement in rotational performance achieved by the flap modification under comparable wind conditions, rather than to perform a direct aerodynamic comparison.

Table 8 summarises the measured shaft speeds for both configurations under different wind conditions. The PF consistently produced higher rotational speeds, recording 19 RPM at 4.5 m/s compared with 14 RPM for the baseline (+ 35.7%). At 5.0 m/s, the PF reached 21.9 RPM versus 18.6 RPM (+ 17.7%), and at 5.5 m/s, the advantage increased to 33 RPM, representing a + 51.3% gain. Given the estimated measurement uncertainty of $\:\pm\:$ 2.5% (derived from instrument accuracy and repeatability), this performance gains significantly exceed the error margin, confirming that the aerodynamic improvements are physically substantial.

These improvements align with the CFD predictions, which showed that the PF configuration enhances Cm and Cp by maintaining stable airflow over the suction surface and suppressing early stall at low TSRs. The observed increase in RPM confirms that the flap modification reduces negative torque regions, improves the lift-to-drag balance, and enables smoother acceleration from rest.

It should be noted that the present experiment was qualitative in scope, intended to verify the improvement trend of the PF design rather than determine absolute performance coefficients. Due to the absence of a wind-tunnel facility and torque measurement instrumentation, experimental Cp and Cm values were not obtained. The measured RPM was therefore used as a practical indicator of aerodynamic enhancement.

Only the 0.5c, 10° PF configuration was tested experimentally. Although CFD results indicated that the 0.015c PGF achieved slightly higher torque (within 5%), the PF was selected for validation due to its simpler geometry, greater structural stability, and easier fabrication. The PGF and GF designs require precise trailing-edge alignment and added stiffness, which are difficult to achieve with small-scale 3D-printed blades.

The PF maintained a clear performance advantage across all tested wind speeds, confirming its ability to improve self-starting and power capture under low-wind conditions. These findings support the CFD-based conclusion that the PF modification strengthens aerodynamic efficiency in small-scale Darrieus turbines. Future work will include wind-tunnel testing with torque measurement to determine the full experimental Cp-TSR characteristics and enable quantitative validation.

**Table 8:** Shaft speed (RPM) comparison of baseline NACA 0015 and 0.5c, 10° PF modification under varying wind speeds, with percentage improvement.

Wind speed (m/s)	NACA 0015 (RPM)	0015 0.5c, 10° PF (RPM)
4.5	14	19 (35.7%)
5.0	18.6	21.9 (17.7%)
5.5	21.8	33 (51.3%)

### Practical design implications

For practical design, the present results suggest clear guidelines for 1–2.5 m diameter Darrieus VAWTs operating in rural and urban winds of 4–10 m/s (Re ≈ 5.4 × 10^4^–2.7 × 10^5^). The 0.5c, 10° PF emerges as the default passive modification. It improves mean Cm by about 30–40% at TSR 0.2–0.5 and increases Cp by up to 49.1% at TSR 1.5, while still providing 4–7% gains at TSR 2.0–2.5. This configuration therefore offers the best compromise between self-starting and rated-speed efficiency and is recommended as the primary option for 1–2.5 m diameter rural VAWTs in this Re range.

The PGF configuration is more specialised. It delivers the highest relative Cp gain at TSR ≈ 1.0 (52.7%) and slightly higher Cm than the PF at TSR 0.8–1.0, but its advantage diminishes and then reverses beyond TSR ≈ 2.0 due to increased drag. PGFs are therefore suited to turbines designed to operate mostly in the start-up and low-TSR regime, where rapid acceleration is prioritised over peak Cp, and where added geometric complexity is acceptable.

Stand-alone GFs (0.02–0.025c at 120°) show modest Cp gains only in a narrow band at moderate TSRs (2.5–3.5) and can reduce performance outside this range. They are only recommended when a PF cannot be integrated (for example, due to manufacturing constraints) and when the design TSR is known to remain near these moderate values. Given the smaller gains, added drag, and fabrication challenges at very small scale, the PF offers the most robust and practically attractive solution.

Overall, for 1–2.5 m diameter Darrieus turbines in low-to-moderate Re conditions, a 0.5c, 10° PF provides a simple, manufacturable, and robust configuration, while PGF and GF layouts are reserved for specific operating TSR windows and more constrained design cases.

## Conclusions

This study provides a systematic aerodynamic assessment of PF, GF, and hybrid PGF configurations applied to the NACA 0015 aerofoil for Darrieus VAWTs. Three principal outcomes emerge from the investigation.

First, the unsteady RANS simulations, supported by prototype measurements, establish a consistent performance benchmark across Re of 5.4 × 10^4^–2.7 × 10^5^ and TSRs of 0.2–4.5. This combined numerical, experimental framework provides a reliable basis for evaluating trailing-edge modifications for small-scale VAWTs.

Second, the 0.5c, 10° PF is identified as the most effective configuration. It delivers 30–40% improvements in mean torque at low TSRs, maintains stable efficiency at higher TSRs, and avoids the drag penalties observed in the PGF beyond TSR ≈ 2.0. In contrast, the PGF enhances initial torque but loses effectiveness as rotational speed increases, confirming its suitability only for specific low-TSR operating conditions.

Third, qualitative prototype testing reinforces the numerical trends. The PF-modified rotor increased shaft speed by 36%, 18%, and 51% at wind velocities of 4.5, 5.0, and 5.5 m/s, respectively, demonstrating the practical validity of the aerodynamic improvements and their relevance for small-scale hybrid wind-solar systems.

For 1–2.5 m diameter Darrieus VAWTs operating at Re ≈ 5.4 × 10^4^–2.7 × 10^5^, the findings support the 0.5c, 10° PF as a robust and manufacturable trailing-edge modification, offering consistent performance gains without added structural or fabrication complexity. GF and PGF configurations should be considered for niche applications requiring enhanced start-up behaviour or operation within narrow TSR ranges.

### Limitations and future work

This study is based on 2D CFD simulations, which do not capture three-dimensional effects such as tip losses, spanwise flow, or full blade-vortex interactions. Transition modelling is not included, which may affect accuracy at low Re. Structural behaviour, including flap-induced stresses and fatigue, is not assessed, and the prototype experiments provide only qualitative verification of aerodynamic trends.

Future work will address these limitations by integrating 3D transient simulations, transition-sensitive turbulence models, and coupled fluid-structure interaction analysis. Long-duration fatigue testing of flap-modified blades will be conducted to evaluate durability under realistic cyclic loading. Active or morphing flap concepts will also be explored to enable real-time aerodynamic adaptation. A techno-economic assessment will be included to quantify performance gains relative to fabrication complexity for small off-grid applications.

## Data Availability

The datasets generated and analysed during the current study are available from the corresponding author upon reasonable request.

## Symbols

*V*_*in*_: Inlet velocity
*R*: Rotor radius
*C*_*p*_: Power coefficient
*Cm*: Torque coefficient

## Greek

$$\:\mu\:$$: Viscosity (Pa.s)
$$\:\omega\:$$: Angular velocity
*ρ*: Air density (kg/m^3^)

## Abbreviations

TSR: Tip speed ratio
VAWT: Vertical axis wind turbine
CFD: Computational fluid dynamics
PGF: Plain gurney flaps
GF: Gurney flap
PF: Plain flap
CFL: Courant–Friedrichs–Lewy

## References

[1] Backwell, B. GWEC’s Global Wind Report 2024. (2024). https://gwec.net/global-wind-report-2024/

[2] Renewable Energy Magazine. Record Year for Wind Energy Shows Momentum, Highlights Need for Policy-Driven Action. (2023). https://www.renewableenergymagazine.com/

[3] Idriss, A. I., Ahmed, R., Atteyeh, H. A., Omar, A. I. & Akinci, T. Techno-economic assessment and wind energy potential of Nagad in Djibouti. *Int. J. Appl. Power Eng.*10.11591/ijape.v13.i1.pp91-101 (2024).

[4] Redchyts, D. et al. Comparison of aerodynamics of vertical-axis wind turbine with single and combine Darrieus and Savonius rotors. *Results Eng.*10.1016/j.rineng.2024.103202 (2024).

[5] Gemayel, D., Abdelwahab, M., Ghazal, T. & Aboshosha, H. Modelling of vertical axis wind turbine using large eddy simulations. *Results Eng.* **18**, 101226 (2023).

[6] Seifi Davari, H., Seify Davari, M. & Kouravand, S. & Kafili Kurdkandi, M. Optimizing the aerodynamic efficiency of different airfoils by altering their geometry at low Reynolds numbers. *Arab. J. Sci. Eng.* 1–36 (2024).

[7] Davari, H. S., Botez, R. M., Davari, M. S., Chowdhury, H. & Hosseinzadeh, H. Blade height impact on self-starting torque for Darrieus vertical axis wind turbines. *Energy Convers. Manag. X* 100814 (2024).

[8] Seifi Davari, H., Seify Davari, M., Botez, R. M. & Chowdhury, H. Advancements in vertical axis wind turbine technologies: A comprehensive review. *Arab. J. Sci. Eng.* 1–48 (2024).

[9] Davari, H. S., Botez, R. M., Davari, M. S., Chowdhury, H. & Hosseinzadeh, H. Numerical and experimental investigation of Darrieus vertical axis wind turbines to enhance self-starting at low wind speeds. *Results Eng.*10.1016/j.rineng.2024.103240 (2024).

[10] Ramlee, M. F., Ramlee, N. & Fazlizan, A. Effect of parametric design in the performance optimization of vertical axis wind turbine: A review. *J. Kejuruter*. 10.17576/jkukm-2023-35(5)-02 (2023).

[11] Cazzaro, D., Bedon, G. & Pisinger, D. Vertical axis wind turbine layout optimization. *Energies*10.3390/en16062697 (2023).

[12] Seifi Davari, H., Kouravand, S., Seify Davari, M. & Kamalnejad, Z. Numerical investigation and aerodynamic simulation of Darrieus H-rotor wind turbine at low Reynolds numbers. *Energy Sources Part. Recover Util. Environ. Eff.* **45**, 6813–6833 (2023).

[13] Guerra, J., Velásquez, L., Rubio-Clemente, A., Jaramillo, L. & Chica, E. Design and optimization of a siphon turbine using the response surface methodology. *Results Eng* 102241 (2024).

[14] Celik, Y. A. Comparative aerodynamic analysis of NACA and NREL aerofoils for Darrieus turbines using CFD. *Int. J. Innov. Eng. Appl.*10.46460/ijiea.1075684 (2022).

[15] Maleki Dastjerdi, S., HormoziNejad, A., Gharali, K. & Nathwani, J. Numerical investigation of VAWT airfoil shapes on power extraction and self-starting purposes. In *Recent Developments in Mathematical, Statistical and Computational Sciences* (eds Kilgour, D. M., Kunze, H., Makarov, R., Melnik, R. & Wang, X.) 383–392 (Springer International Publishing, 2021).

[16] Mohamed, M. H. Criticism study of J-Shaped Darrieus wind turbine: performance evaluation and noise generation assessment. *Energy* **177**, 367–385 (2019).

[17] Zhang, L. et al. Influences of trailing edge split flap on the aerodynamic performance of vertical axis wind turbine. *Energy Sci. Eng.* **9**, 101–115 (2021).

[18] Singh, I. Effect of plain flap over the aerodynamic characteristics of airfoil NACA 66 -015. *Int. J. Innov. Sci. Res. Technol.* **2**, 353–365 (2017).

[19] Abed, A. Computational analysis of SD7037 airfoil with plain flap. *Arch. Mech. Eng.*10.24425/ame.2024.151337 (2024).

[20] Kaya, A. F. Investigation of a rib structure effect on the aerodynamic performance of a plain flapped symmetrical airfoil. *J. Polytech.*10.2339/politeknik.1159822 (2023).

[21] Parluhutan, Y. M., Fahrudin, F. & Rhakasywi, D. Investigating the impact of plain flap as lift enhancement on symmetrical airfoils. *Int. J. Mar. Eng. Innov. Res.* **9**, (2024).

[22] Eltayeb, W., Somlal, J., Singh, A. R. & Alsaif, F. Enhancing Darrieus wind turbine performance through varied plain flap configurations for the solar and wind tree. 1–22 (2024). 10.1038/s41598-024-81853-6 PMC11612441 39623204

[23] Li, Y. C., Wang, J. J., Tan, G. K. & Zhang, P. F. Effects of gurney flaps on the lift enhancement of a cropped nonslender delta wing. *Exp. Fluids*. **32**, 99–105 (2002).

[24] Xie, Y. H., Jiang, W., Lu, K. & Zhang, D. Numerical investigation into energy extraction of flapping airfoil with gurney flaps. *Energy* **109**, 694–702 (2016).

[25] Yan, Y., Avital, E., Williams, J. & Cui, J. Performance improvements for a vertical axis wind turbine by means of gurney flap. *J. Fluids Eng.* **142**, 21205 (2020).

[26] Bianchini, A., Balduzzi, F., Di Rosa, D. & Ferrara, G. On the use of gurney flaps for the aerodynamic performance augmentation of Darrieus wind turbines. *Energy Convers. Manag*. **184**, 402–415 (2019).

[27] Mousavi, M., Masdari, M. & Tahani, M. Power performance enhancement of vertical axis wind turbines by a novel gurney flap design. *Aircr. Eng. Aerosp. Technol.* **94**, 482–491 (2022).

[28] Pambudi, F. K., Tjahjana, D. D. D. P. & Santoso, B. Experimental study of gurney flap on Darrieus wind turbine performance. *Int. J. Appl. Sci. Technol. Eng.*10.24912/ijaste.v1.i1.269-274 (2023).

[29] Chen, L., Yang, P., Zhang, B. & Chen, L. Aerodynamic enhancement of Vertical-Axis wind turbines using plain and serrated gurney flaps. *Appl. Sci.* **13**, 12643 (2023).

[30] Chakroun, Y. & Bangga, G. Aerodynamic characteristics of airfoil and vertical axis wind turbine employed with gurney flaps. *Sustainability*. **13**, 4284 (2021).

[31] Carrigan, T. J., Dennis, B. H., Han, Z. X. & Wang, B. P. Aerodynamic shape optimization of a vertical-axis wind turbine using differential evolution. *Int. Sch. Res. Not.* (2012).

[32] Abdalrahman, G., Melek, W. & Lien, F. S. Pitch angle control for a small-scale Darrieus vertical axis wind turbine with straight blades (H-Type VAWT). *Renew. Energy*. **114**, 1353–1362 (2017).

[33] Gosselin, R., Dumas, G. & Boudreau, M. Parametric study of H-Darrieus vertical-axis turbines using CFD simulations. *J. Renew. Sustain. Energy* **8**, (2016).

[34] Singh, M. & Santoso, S. *Dynamic Models for Wind Turbines and Wind Power Plants* (2011).

[35] Castillo, O. C., Andrade, V. R., Rivas, J. J. R. & González, R. O. Comparison of power coefficients in wind turbines considering the tip speed ratio and blade pitch angle. *Energies* **16**, 2774 (2023).

[36] Rezaeiha, A., Montazeri, H. & Blocken, B. Towards accurate CFD simulations of vertical axis wind turbines at different tip speed ratios and solidities: guidelines for azimuthal increment, domain size and convergence. *Energy Convers. Manag*. **156**, 301–316 (2018).

[37] Mazarbhuiya, H. M. S. M., Biswas, A. & Sharma, K. K. Effect of blade attachments on the performance of an asymmetric blade H-Darrieus turbine at low wind speed. *Energy Sources Part. Recover Util. Environ. Eff* 1–18 (2020).

[38] Sengupta, A. R., Biswas, A. & Gupta, R. Comparison of low wind speed aerodynamics of unsymmetrical blade H-Darrieus rotors-blade camber and curvature signatures for performance improvement. *Renew. Energy*. **139**, 1412–1427 (2019).

[39] Reddy, K. U., Deb, B. & Roy, B. A numerical and experimental study on the performance of a conventional H-Darrieus wind rotor with auxiliary blades. *Ocean. Eng.* **280**, 114697 (2023).

[40] Rezaeiha, A., Montazeri, H. & Blocken, B. Characterization of aerodynamic performance of vertical axis wind turbines: impact of operational parameters. *Energy Convers. Manag*. **169**, 45–77 (2018).

[41] Bravo, R., Tullis, S. & Ziada, S. Performance testing of a small vertical-axis wind turbine. In *Proceedings of the 21st Canadian Congress of Applied Mechanics*, 3–7 (2007).

[42] Chen, C. C. & Kuo, C. H. Effects of pitch angle and blade camber on flow characteristics and performance of small-size Darrieus VAWT. *J. Vis.* **16**, 65–74 (2013).

[43] Barnes, A., Marshall-Cross, D. & Hughes, B. R. Towards a standard approach for future vertical axis wind turbine aerodynamics research and development. *Renew. Sustain. Energy Rev.* **148**, 111221 (2021).

[44] Le Fouest, S. & Mulleners, K. Optimal blade pitch control for enhanced vertical-axis wind turbine performance. *Nat. Commun.* **15**, 2770 (2024). 38553502 10.1038/s41467-024-46988-0 PMC10980684

[45] Yang, P., Chen, L., Zhang, B. X. & He, C. C. Performance improvements of vertical axis wind turbines with modified gurney flaps. *J. Phys. Conf. Ser.* **2707**, 12077 (2024).

[46] Abdallah, A., William, M. A., Moharram, N. A. & Zidane, I. F. Boosting H-Darrieus vertical axis wind turbine performance: A CFD investigation of J-Blade aerodynamics. *Results Eng.* 106358 (2025).

[47] Roshan, M. Y., Khaleghinia, J., Nimvari, M. E. & Salarian, H. Performance improvement of Darrieus wind turbine using different cavity layouts. *Energy Convers. Manag*. **246**, 114693 (2021).

[48] Yin, R. Impacts of external airfoil flaps on the S809 airfoil. *Sci. Rep.* **15**, 31797 (2025). 40877403 10.1038/s41598-025-16954-x PMC12394577

[49] Anderson, J. *EBOOK: Fundamentals of Aerodynamics (SI Units)* (McGraw hill, 2011).

[50] Wang, J. J., Li, Y. C. & Choi, K. S. Gurney flap—Lift enhancement, mechanisms and applications. *Prog. Aerosp. Sci.* **44**, 22–47 (2008).

[51] Brown, L. & Filippone, A. Aerofoil at low speeds with gurney flaps. *Aeronaut. J.* **107**, 539–546 (2003).

[52] Alber, J. et al. Aerodynamic effects of gurney flaps on the rotor blades of a research wind turbine. *Wind Energy Sci.* **5**, 1645–1662 (2020).

[53] Syawitri, T. P., Yao, Y., Yao, J. & Chandra, B. Geometry optimisation of vertical axis wind turbine with gurney flap for performance enhancement at low, medium and high ranges of tip speed ratios. *Sustain. Energy Technol. Assess*. **49**, 101779 (2022).

[54] Chen, L., Xu, J. & Dai, R. Numerical prediction of switching gurney flap effects on straight bladed VAWT power performance. *J. Mech. Sci. Technol.* **34**, 4933–4940 (2020).

[55] Kord, K. & Bazargan, M. Numerical investigation on J-shaped straight-bladed darrieus vertical axis wind turbines equipped with gurney flaps. *Int. J. Energy Res.* **2024**, 8992210 (2024).

